# Supplementary material for: Thermodynamics of diamond formation from hydrocarbon mixtures in planets
Source: Nat Commun. 2023 Feb 27;14:1104. doi: 10.1038/s41467-023-36841-1 (PMC9968715; doi:10.1038/s41467-023-36841-1)
Supplement: Supplementary file 1 — Supplementary Information [file 41467_2023_36841_MOESM1_ESM.pdf]

Thermodynamics of diamond formation from hydrocarbon mixtures in planets
Supplementary Information

Bingqing Cheng,[1,*] Sebastien Hamel,[2] and Mandy Bethkenhagen[3,1]

[1]*The Institute of Science and Technology Austria, Am Campus 1, 3400 Klosterneuburg, Austria*

[2]*Lawrence Livermore National Laboratory, Livermore, California 94550, USA*

[3]*École Normale Supérieure de Lyon, Université Lyon 1, Laboratoire de Géologie de Lyon, CNRS UMR 5276, 69364 Lyon Cedex 07, France*

(Dated: January 28, 2023)

* bingqing.cheng@ist.ac.at

CONTENTS

I. Details on the DFT calculations 3

A. Convergence tests for DFT 3

B. Influence of the Fermi smearing 4

II. DFT MD calculations 5

III. Details on constructing the MLP 7

A. Training set 7

B. Training 8

C. Validation 9

IV. Benchmarks of the MLP 10

A. NVT simulation of pure liquid carbon 10

B. NVT simulation of diamond 12

C. NVT simulation of pure H 14

D. NVT simulation of CH_4 16

E. NVT simulation of CH_2 23

F. NVT simulation of C_2H 28

G. Enthalpy of different solid carbon polymorphs 32

V. Simulations using the MLP 34

A. MLP MD simulations 34

B. NPT simulations of diamond and pure liquid carbon 34

C. Chemical potentials of pure carbon systems 35

D. Distribution of C-C bonds in CH_4 36

E. Chemical potential of C in C/H mixtures 36

F. Hydrocarbon crystals 38

VI. Carbon contents of planets and stars 40

A. Atmosphere 40

B. Neptune and Uranus interior models 41

C. White Dwarfs 41

I. DETAILS ON THE DFT CALCULATIONS

We used the planewave code VASP [1–3] for production DFT calculations using the exchange-correlation functional by Perdew, Burke, and Ernzerhof (PBE) [4] and sampled the Brillouin zone using a k-point grid resolution of at least 0.2 $Å^{-1}$. The planewave energy cutoff is set to 1000 eV and we used the C_h and H_h PBE projector augmented wave (PAW) potentials from the VASP library. These consist of the hard version of the PAW potentials. The accuracy of this carbon PAW potential at high pressure has been the object of a study by Benedict et al. [5]. These authors reported that the 4-electron hard PAW from the VASP library compared well with a 6-electron (all-electron) PAW potential up to densities of 60 g/cm^3, corresponding to carbon-carbon distance of 1.3 atomic units (0.7 Å).

A. Convergence tests for DFT

The convergence of our DFT calculations used for the evaluation of the training set was extensively tested. In particular, we paid close attention to the planewave energy cutoff and the k-point grid resolution, which are the most important parameters to ensure the targeted accuracy of the training set. We chose a diverse set of configurations to test both the planewave energy cutoff and the k-point grid resolution, including an isolated carbon atom, a carbon dimer, amorphous carbon between 3 g/cm^3 and 6.79 g/cm^3 and a high pressure phase of carbon (simple cubic). We also included liquid-like C/H structures with carbon concentrations of 0.008, 0.25, 0.67 and 0.89. The chosen configurations span the densities, pressure and stoichiometries of interest and have no electronic band gap hence they are particularly sensitive to the k-point grid resolution. We found that Γ-centered k-point grids with a "KSPACING" parameter of 0.2 $Å^{-1}$ are converged to better than 0.1% in pressure and 1 meV/atom in energy compared to a k-spacing of 0.1 $Å^{-1}$. A planewave energy cutoff of 1000 eV is sufficient to reach 1% in pressure and 4 meV/atom in energy compared to 1500 eV calculations.

B. Influence of the Fermi smearing

In this study we use an approximation to finite-temperature DFT. The Kohn-Sham electrons are assigned a temperature through a Fermi smearing of the orbital occupation numbers while the exchange-correlation (XC) functional is set to the zero-K PBE functional. A finite-temperature extension of the PBE functional is not yet available and so the impact of this approximation is not known. However, in the case of the local density approximation (LDA) the zero-K XC approximation was shown to lead to very small changes to the equation of state for temperatures at or below 10000 K (less than a 1 % pressure difference) compared to the temperature-dependent LDA XC functional for both hydrogen [6] and carbon [7].

The forces in DFT are calculated based on the electronic free energy

$$F_{\mathrm{el}} = E_{\mathrm{el}} - T_{\mathrm{el}} S_{\mathrm{el}}, \tag{1}$$

where E_{el} and T_{el} are the internal energy and the temperature of the electrons, respectively. The Kohn-Sham electronic entropy S_{el} in the finite-temperature Fermi smearing approach is calculated according to

$$S_{\mathrm{el}} = \sum_{nk} k_B (\ln(f_{nk}) + \ln(1 - f_{nk})), \tag{2}$$

where k_B is the Boltzmann constant and f_{nk} denotes the occupation number of each electronic band n at k-point k set according to the Fermi distribution:

$$f_{nk} = \frac{1}{\exp\left(\frac{\epsilon_{nk}-\mu}{k_B T_{el}} + 1\right)}, \tag{3}$$

where μ is the electronic chemical potential and ϵ_{nk} the energy of each state. T_{el} is the electronic temperature. In Fig. S1 we benchmark the change in forces as a function of carbon concentration and electronic temperature between 300 K and 9000 K. We calculate the root mean square deviation (RMSD) of the difference between the forces at $T_{\mathrm{el}} = T$ and $T_{\mathrm{el}} = 300\ K$. We find that the effect is composition dependent in this range. The temperature dependence is small for temperatures below 4000 K (RMSD < 0.1 eV/Å) but becomes important at higher temperature: RMSD ~ 0.3 eV/Å at 9000 K for pure carbon (χ_C=1). To take into account the impact of the thermal excitation of the electronic subsystem, for carbon and C/H mixtures we set T_{el} equal to the average ionic temperature during the DFT MD calculations as well as in the reference calculations used to train and test the MLP.

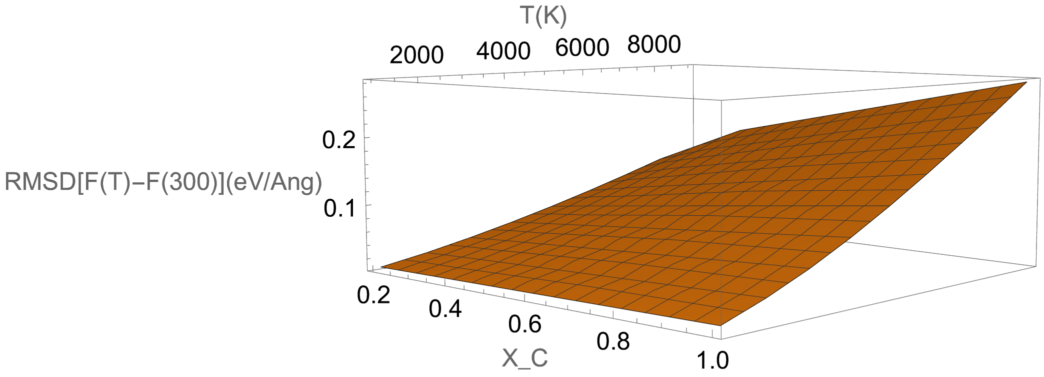

FIG. S1. Influence of the Fermi smearing on the forces at a pressure of about 300 GPa. We show here the root mean square deviation (RMSD) of the difference between the forces calculated using a Fermi smearing at a temperature T and at 300 K, as a function of temperature and for three compositions: C, CH_2 and CH_4.

II. DFT MD CALCULATIONS

Born-Oppenheimer DFT Molecular Dynamics (DFT MD) is the workhorse of high pressure equation-of-state calculations and has shown good agreement with several experiments on hydrocarbons and other systems [8–10] for measured thermodynamic properties in particular for Hugoniot curves in pressure-density space and in pressure-temperature space when the temperature is inferred with pyrometry. We performed DFT MD simulations for the C/H system with various compositions to generate a variety of configurations to train the MLP and to create a data basis of thermodynamic, structural, and diffusive properties to benchmark our results obtained with the MLP. The DFT MD simulations were also performed with VASP [1–3] and used the same DFT parameters as described in section I except for the k-point sampling. The Brillouin zone was sampled at the Baldereschi Mean Value point, since MD runs typically require a less dense k-point grid [11]. The temperature was controlled using a Nosé-Hoover thermostat [12]. Further simulation parameters and considered thermodynamic conditions for each system are specified in the following.

a. DFT MD of pure C liquid This data set consists of simulations of 64 carbon atoms, starting from a liquid configuration, for temperatures between 6000 K and 10000 K and densities between 3 g/cm^3 and 8 g/cm^3 (every 0.1 g/cm^3) yielding pressures between 45 GPa and 1474 GPa. The simulations were run with a timestep of 0.5 fs. The MD trajectories were generated using simulations lengths of at least 10 ps.

b. DFT MD of diamond A second data set for pure carbon was generated for the diamond phase. This data set also consists of simulations of 64 carbon atoms, this time starting from a solid (diamond) configuration for temperatures between 2000 K and 10000 K and densities between 3.7 g/cm^3 and 6.5 g/cm^3 (every 0.2 g/cm^3). We also included a simulation at 3.52 g/cm^3 and 300 K as well as simulations at 6.889 g/cm^3 and temperatures between 6000 K and 10000 K. The pressure range spanned by these simulations is between 4 GPa and 1000 GPa. We used a timestep of 0.5 fs. The MD trajectories were generated using simulations lengths of at least 10 ps

c. DFT MD of CH_4 This data set is expanded based on earlier DFT MD calculations that primarily targeted the equation of state of high-pressure methane [13]. In total we considered 141 different density-temperature points, which we reanalyzed and partially extended in simulation length. The thermodynamic conditions cover densities between 0.60 g/cm^3 and 4.50 g/cm^3 yielding pressures between 3 GPa and 1440 GPa along 11 isotherms between 1000 K and 14000 K. The simulation cell contains 54 methane molecules that were initially placed on a bcc lattice. The simulations were run with a timestep of 0.25 fs. The MD trajectories were generated using simulations lengths of at least 10 ps.

d. DFT MD of other C/H mixtures We performed an extensive amount of DFT MD simulations for CH_{16} (with 27 C and 432 H), CH_8 (with 36 C and 288 H), CH_2 (with 48 C and 96 H), CH (with 48 C and 48 H), C_2H (with 48 C and 24 H), and C_4H (with 64 C and 16 H) mixtures. The density grid was chosen to roughly cover a pressure range between 50 GPa and 600 GPa for each composition. We considered the following densities for CH_{16}: 0.75 g/cm^3, 1.05 g/cm^3, 1.35 g/cm^3, 1.65 g/cm^3, 1.85 g/cm^3, 2.15 g/cm^3; for CH_8: 0.95 g/cm^3, 1.30 g/cm^3, 1.70 g/cm^3, 2.05 g/cm^3, 2.30 g/cm^3, 2.70 g/cm^3; for CH: 2.30 g/cm^3, 2.75 g/cm^3, 3.35 g/cm^3, 3.80 g/cm^3, 4.20 g/cm^3, 4.80 g/cm^3; for CH_2: 1.75 g/cm^3, 2.20 g/cm^3, 2.75 g/cm^3, 3.25 g/cm^3, 3.60 g/cm^3, 4.15 g/cm^3; for C_2H: 2.65 g/cm^3, 3.10 g/cm^3, 3.75 g/cm^3, 4.20 g/cm^3, 4.60 g/cm^3, 5.25 g/cm^3; for C_4H: 2.85 g/cm^3, 3.40 g/cm^3, 4.00 g/cm^3, 4.50 g/cm^3, 4.90 g/cm^3, 5.60 g/cm^3. For the carbon-rich mixtures C_2H and C_4H, we simulated the six different densities at 4000 K, 6000 K, and 8000 K; for all the other mixtures, we simulated six different densities at 3000 K, 4000 K, 6000 K, and 8000 K. All simulations were run for at least 7.5 ps at 8000 K and 12.5 ps for all other temperatures with a timestep size of 0.25 fs.

III. DETAILS ON CONSTRUCTING THE MLP

To generate flexible and dissociable MLPs for high-pressure C/H mixtures, we employed an artificial neural network architecture built according to the framework of Behler and Parrinello [14], and used the N2P2 code [15]. In this framework, the total energy of the system is expressed as the sum of the individual contributions from atom-centered environments that encompass the relative coordinates of all neighboring atoms inside a cutoff radius. We used a cutoff radius of 10 Bohr. The same cutoff was also used for a previous MLP built for hydrogen, and a recent GAP model for carbon used 4.2Å [16] and 3.1Å [17]. To remove the rotational as well as the permutation variances of atomic coordinates, we selected 123 Behler-Parrinello symmetry functions (SFs) for carbon and 107 SFs for hydrogen atoms to describe the atomic environments, according to the correlations between the values of the SFs and the magnitude of forces on central atoms. The values of the SFs are then used as input vectors for the atomic neural networks that contain two hidden layers with 20 nodes each, yielding the atomic energy contributions. Finally, the total energy is a sum over the outputs of all individual atomic neural networks, and analytic gradients for the calculation of forces are readily available.

We constructed two versions of the MLPs (V1 and V2). V1 was used for all the production simulations with carbon fractions $\chi_C \geq 0.2$ (CH_4 or more carbon-rich components), and V2 was used for all the other production simulations with $\chi_C < 0.2$. The behaviors of the two MLPs for systems with $\chi_C \geq 0.2$ are very similar, but V2 is more accurate for dilute carbon solutions. Both versions of the MLPs are provided in the Supplementary Information.

A. Training set

The training set for the MLP V1 has 84,657 configurations with a total of 7,133,531 atoms. For the MLP V2, the training set was expanded by including another 7,528 configurations with a total of 1,773,051 atoms.

a. FPS selection from DFT MD simulations The 6,490 most structurally diverse configurations were extracted from all the DFT MD trajectories of 54 CH_4 molecules using a farthest point sampling (FPS) algorithm. The ASAP code [18] was used in the visualization and the sparsification of the data set.

b. High-pressure hydrogen We added 48,679 snapshots of pure hydrogen configurations each with 8 to 128 atoms, which have previously served in training a recent MLP of pure hydrogen [19].

c. Low and high-pressure carbon We used all the carbon structures from the GAP20 data set [16], which contains 6,088 configurations with from 1 to 644 atoms. The GAP20 set includes a large variety of carbon structures/molecules with single, aromatic, double bonds: cubic/hexagonal diamond, graphite, graphane, carbon nanotubes, fullerenes, amorphous carbon, and defected structures [16]. The GAP20 is regarded to well-capture the chemistry of carbon, because it has been thoroughly benchmarked using metrics such as formation energies, radial distribution functions and the torsional energies of carbon bonds [16]. We also added 2,000 snapshots of pure liquid carbon configurations of 64 atoms selected using FPS from DFT MD trajectories, and another 2,000 solid structure of 72 or 108 atoms obtained from random searches and then adding small random displacements.

d. Random searches We included 17,500 structures with variable compositions generated using random crystal structure searches [20] using the AIRSS code employing different fits of the MLP. The system size of the structures in this set ranges from 32 to 254 atoms, and the simulation cell has either cubic or triclinic shapes.

e. Active learning We performed active learning in order to refine the training set. The snapshots of atomic positions were generated from replica exchange molecular dynamics runs at different temperatures. Subsequently, the structures with large variance of the predicted energies from 4 different fits of the MLP were selected. 1,900 structures each with 64-432 atoms of composition C, CH, CH_2, CH_3, C_2H, C_3H were included this way.

f. Extra structures for training MLP V2 To improve the description of the MLP for low carbon concentration mixtures, we used 7,528 structures with variable C/H ratios generated from MD simulations. This set was only used for training MLP V2.

B. Training

For training the MLP V1, the resulting root mean squared errors (RMSE) of the energies in the training and test sets are 43 meV/atom and 42 meV/atom, respectively, and the RMSE values of the forces in the training and test sets are 865 meV/Å and 767 meV/Å, respectively.

For training the MLP V2, the RMSE of the energies in the training and test sets are 42 meV/atom

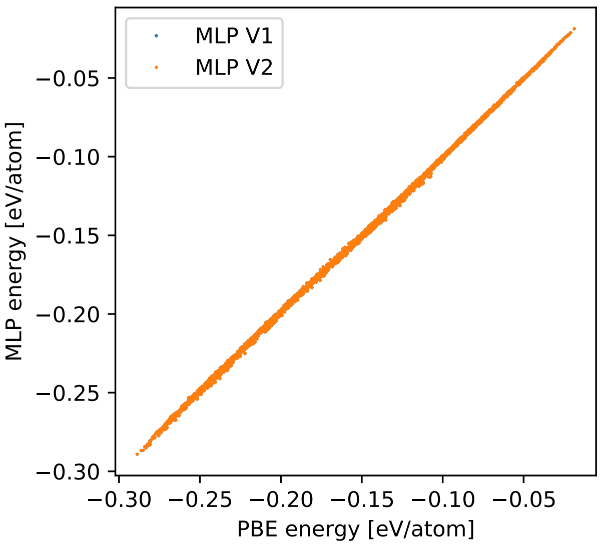

FIG. S2. A comparison between the MLP and the PBE energies for the structures in the validation set.

and 45 meV/atom, respectively, and the RMSE values of the forces in the training and test sets are 922 meV/Å and 800 meV/Å, respectively.

C. Validation

To validate the MLPs, we generated a very diverse set of C/H structures with variable compositions (73-380 total number of atoms in each configuration, carbon atomic fraction from about 1% to 94%). This set has a total of 3,884 structures, and was generated by running MLP NPT simulations at T =2500 K, 3000 K, 4000 K, 6000 K, and pressures between 10 GPa to 600 GPa. We then computed the PBE energies for these configurations and compared with the MLP energies, and the parity plot is shown in Fig. S2. For MLP V1, the resulting RMSE of the energies are 35 meV/atom, and the RMSE values of the forces are 827 meV/Å. For MLP V2, the resulting RMSE of the energies are 23 meV/atom, and the RMSE values of the forces are 807 meV/Å. These errors are smaller than the training/testing errors of the MLPs, meaning that both version of the MLPs can describe these diverse configurations extremely well. The MLP V2 is more accurate, which is due to a better description of the configurations with low carbon concentrations.

IV. BENCHMARKS OF THE MLP

We have performed extensive benchmarks of the MLP by comparing with DFT results based on the PBE exchange-correlation functional, including the comparison of equations of state (EOS), radial distribution functions, diffusion coefficients, vibrational density of states (vDOS), and the enthalpies of solid structures. The details of the DFT MD simulations for pure carbon and the different C/H mixtures including methane are provided in Sec. II, and the DFT MD results for pure hydrogen were taken from Ref. [19]. The NVT simulations using the MLP were carried out using LAMMPS [21] interfaced with N2P2 [22]. The simulation setup of the MLP MD was selected to closely resemble the DFT MD runs: same initial configurations, same thermodynamic conditions, same time step size and simulation length. This section provides the detailed benchmark data.

We performed the benchmark based on the systems of liquid carbon, diamond, liquid hydrogen, CH_{16}, CH_8, CH_4, CH_2, CH, C_2H, and C_4H mixtures. For all these systems, we covered a wide range of temperature and pressure conditions (50 GPa-600 GPa and beyond). Here we present the benchmark of liquid carbon, diamond, liquid hydrogen, CH_4, CH_2, and C_2H, and the rest of the benchmark is provided in the SI repository.

A. NVT simulation of pure liquid carbon

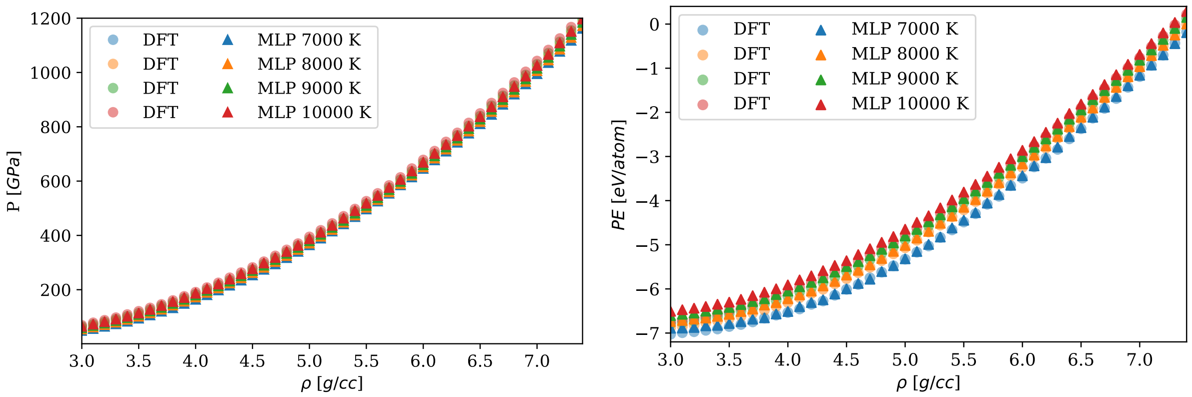

FIG. S3. Pressure (left panel) and potential energy (right panel) of pure liquid carbon computed from equilibrium MD simulations in the NVT ensemble with a system size of 64 carbon atoms. The solid triangular symbols are the results from the MLP, and the semi-transparent circles are from PBE DFT.

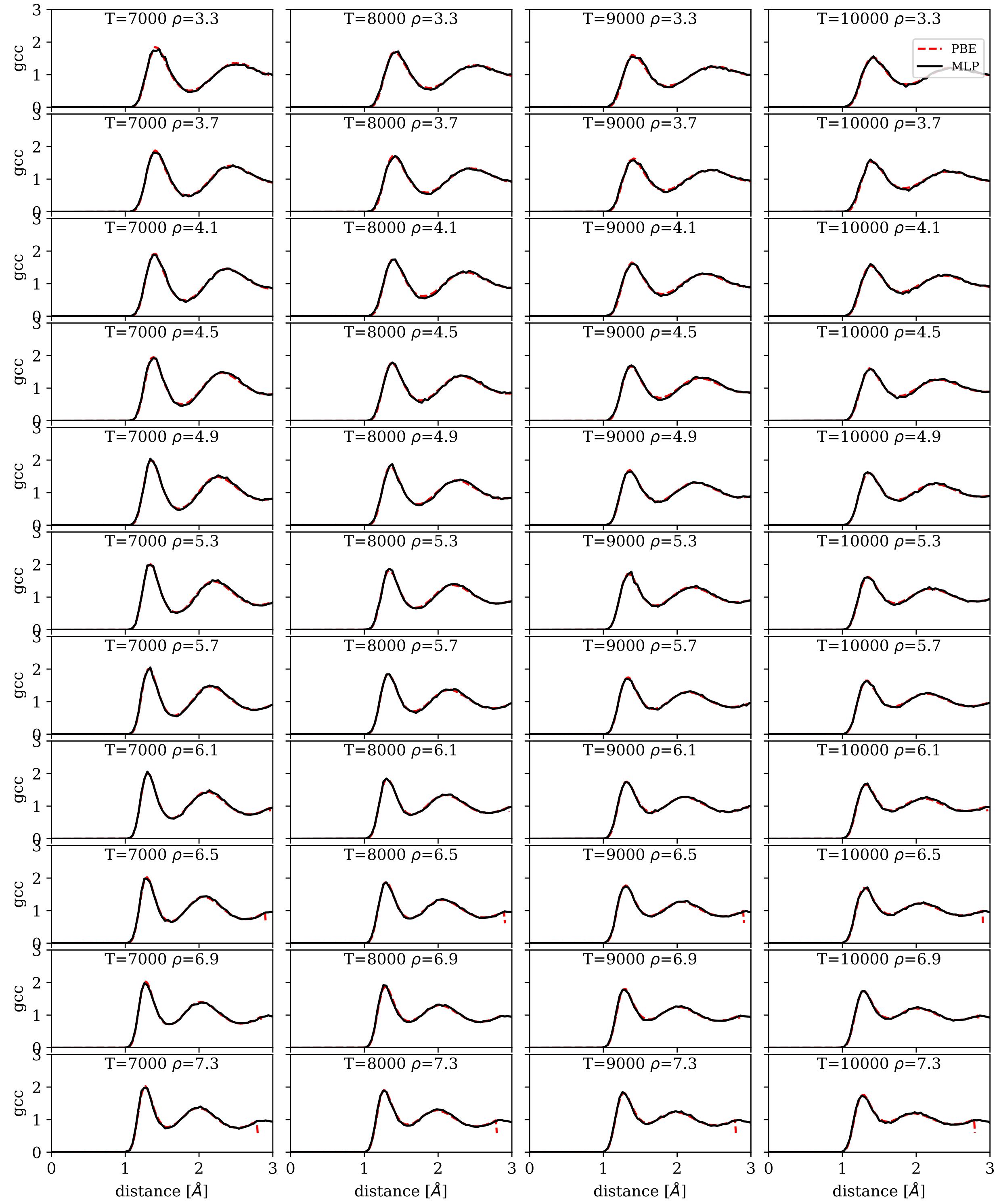

FIG. S4. Carbon-carbon radial distribution functions gcc of pure liquid carbon computed from equilibrium MD simulations in the NVT ensemble with a system size of 64 carbon atoms. The solid black lines are the results from the MLP, and the dashed red lines are from PBE DFT.

The MLP MD data set consists of simulations of 64 carbon atoms starting from a liquid configuration for temperatures between 7000 K and 10000 K and densities between 3.3 g/cm^3 and 7.3 g/cm^3, yielding pressures between about 45 GPa and 1100 GPa. Fig. S3 compares the liquid carbon EOS obtained from the MLP and the DFT NVT simulations, while Fig. S4 shows the respective carbon-carbon radial distribution functions. We also performed the simulations at 6000 K. However, at 6000 K, in both MLP and PBE NVT MD simulations some systems have solidified, so it is not meaningful to compare these simulations.

B. NVT simulation of diamond

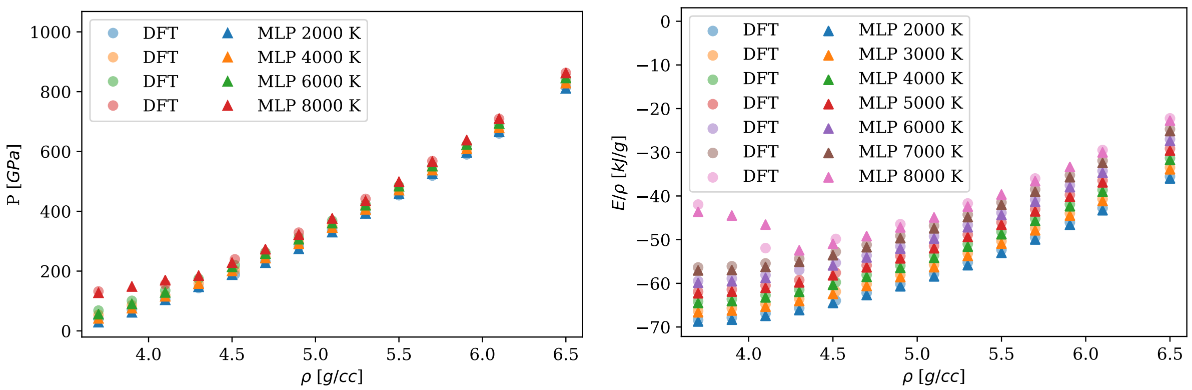

FIG. S5. Pressure (left panel) and equilibrium energy per unit mass (E/ρ) (right panel) of pure diamond computed from equilibrium molecular dynamics simulations in the NVT ensemble with a system size of 64 carbon atoms. The solid triangular symbols are the results from the MLP, and the semi-transparent circles are from PBE DFT.

This data set consists of simulations of 64 carbon atoms, starting from a diamond configuration for temperatures between 2000 K and 8000 K and densities between 3.7 g/cm^3 and 6.5 g/cm^3, yielding pressures between 40 GPa and 1000 GPa. Fig. S5 shows the comparison of EOS from the MLP and the PBE DFT NVT simulations. Both DFT MD and MLP MD simulations of diamond at 8000 K and low densities have melted, with a different onset of melting time during the MD runs. Fig. S6 shows the benchmark results for the C-C radial distribution functions.

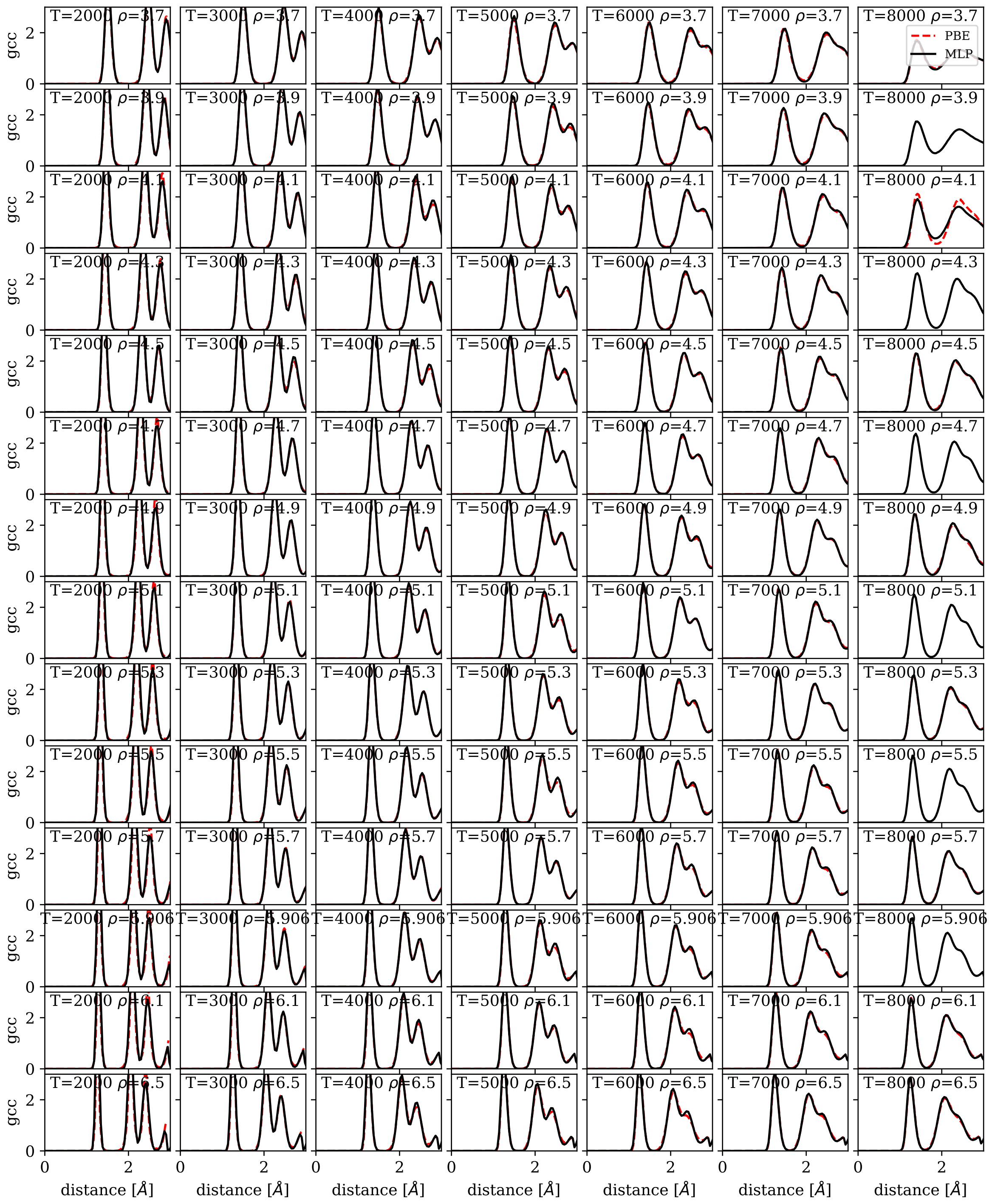

FIG. S6. Carbon-carbon radial distribution functions gcc of pure diamond computed from equilibrium MD simulations at the NVT ensemble with a system size of 64 carbon atoms. The solid black lines are the results from the MLP, and the dashed red lines are from PBE DFT.

C. NVT simulation of pure H

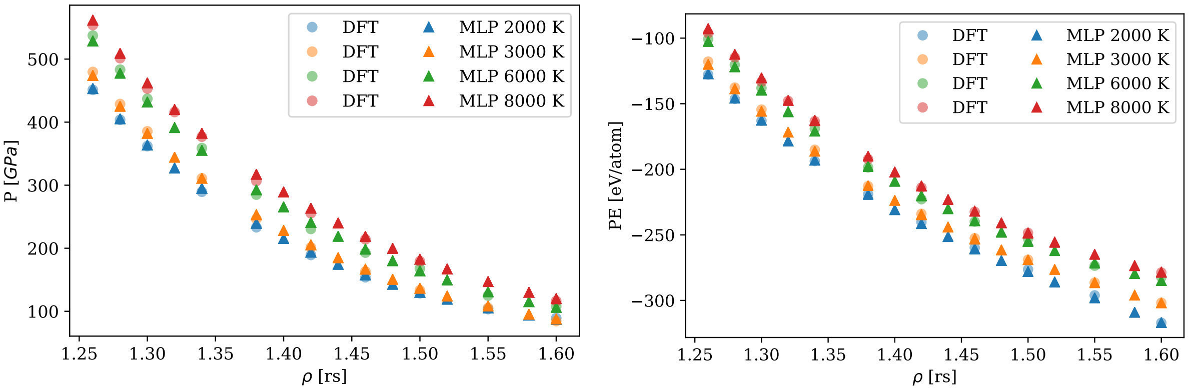

FIG. S7. Pressure (left panel) and potential energy (right panel) of pure liquid hydrogen computed from equilibrium MD simulations in the NVT ensemble with a system size of 128 hydrogen atoms. The solid triangular symbols are the results from the MLP, and the semi-transparent circles are from PBE DFT.

The PBE DFT simulations at the NVT ensemble were taken from Ref. [19]. The densities ranging from $r_s = 1.26$ to $r_s = 1.60$ (i.e. 1.348-0.658 g/mL), correspond to a pressure range of about 80 GPa to 550 GPa. The density is expressed in terms of the Wigner-Seitz radius r_s, as customary in the studies of high-pressure hydrogen. r_s is the radius of a sphere whose volume is equal to the volume per atom in the units of the Bohr radius. The density in this unit can be converted to g/mL via the relationship $\rho[\mathrm{g/mL}] = 2.6966/(\rho[r_s])^3$. The system size is 128 H atoms. We considered temperatures between 2000 K and 8000 K. Fig. S7 shows the comparison of EOS from the MLP and the PBE DFT NVT simulations and Fig. S8 shows the H-H radial distribution functions.

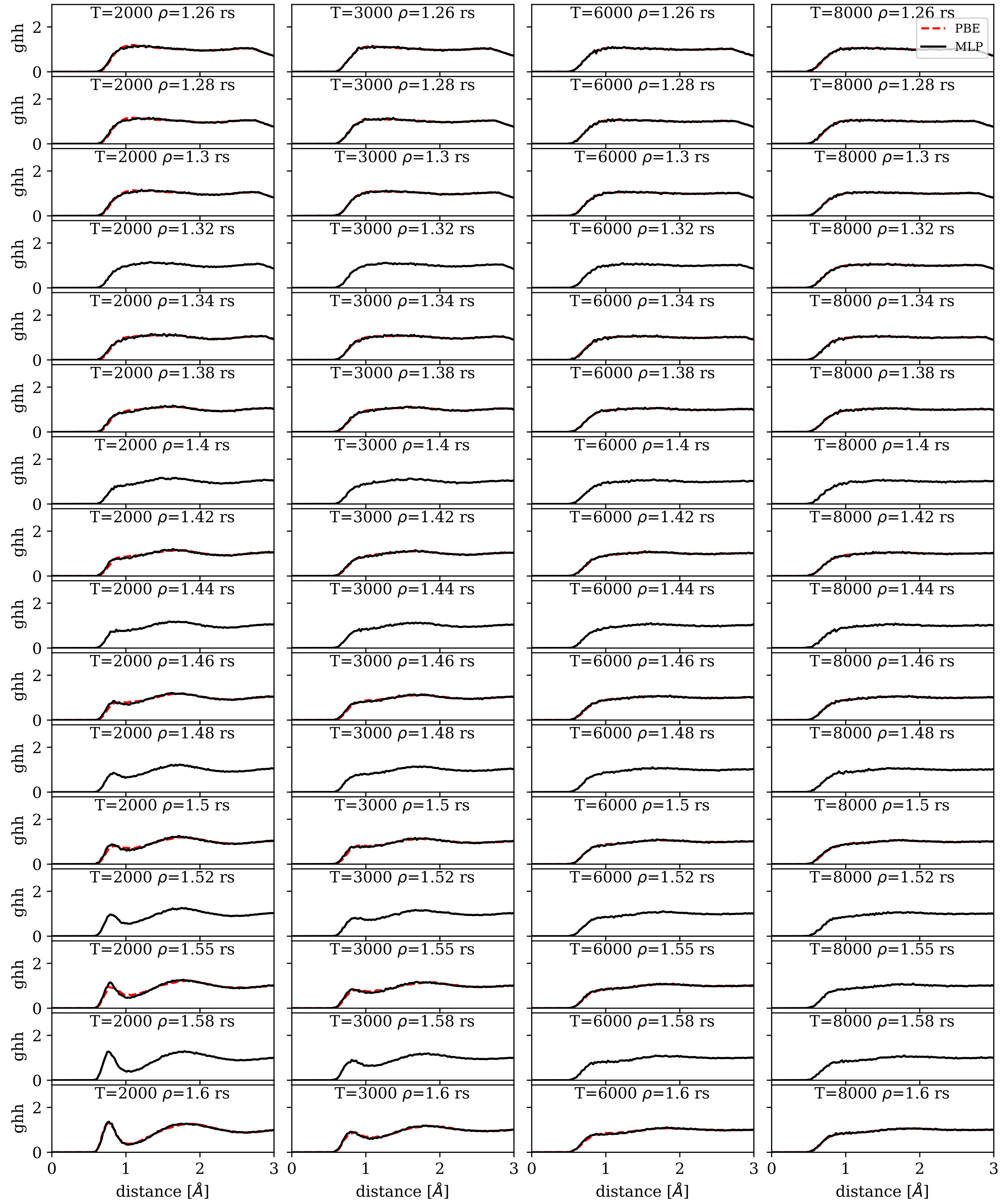

FIG. S8. Hydrogen-hydrogen radial distribution functions ghh of pure hydrogen system computed from equilibrium MD simulations at the NVT ensemble with a system size of 128 hydrogen atoms. The solid black lines are the results from the MLP, and the dashed red lines are from PBE DFT.

D. NVT simulation of CH_4

We performed NVT simulations for systems of 54 CH_4 molecules using the MLP. The initial configuration has 54 methane molecules with ordered orientations placed on a bcc lattice. The choice of this initial configuration was because the same was used in the reference DFT MD simulations. The equations of state and the diffusion coefficients are plotted in Fig. S9. The C-C, C-H and H-H radial distribution functions are plotted in Fig. S10, Fig. S11, and Fig. S12, respectively. The vibrational density of states are plotted in Fig. S13. The thermodynamic conditions cover densities between 0.60 g/cm^3 and 3.50 g/cm^3, and temperatures between 1000 K and 8000 K, corresponding to pressures between about 20 GPa and 700 GPa. At $T \leq 3000$ K, the systems are not ergodic: due to the poor choice of the initial configuration of the bcc lattice, several systems got stuck in this local minima during the short MD simulations and did not melt. As such, here we only compare the simulation results at $T \geq 4000$ K.

From the C-C RDFs, it can be seen that the first neighbor shell, i.e. the first minima in the RDFs, is close to 1.6 Å across a wide range of conditions. Therefore, in the main text we use 1.6 Å as the cutoff distance for identifying C-C bonds. In fig. S14 we show the comparison between the the C-C and C-H bond lifetimes predicted by PBE DFT and the MLP. At 1.5 g/mL, 3000 K there is no DFT data for the C-C bond lifetime because there was not sufficient C-C formation and breaking in the DFT MD simulation. Overall, the MLP reproduces the DFT MD bond lifetimes extremely well.

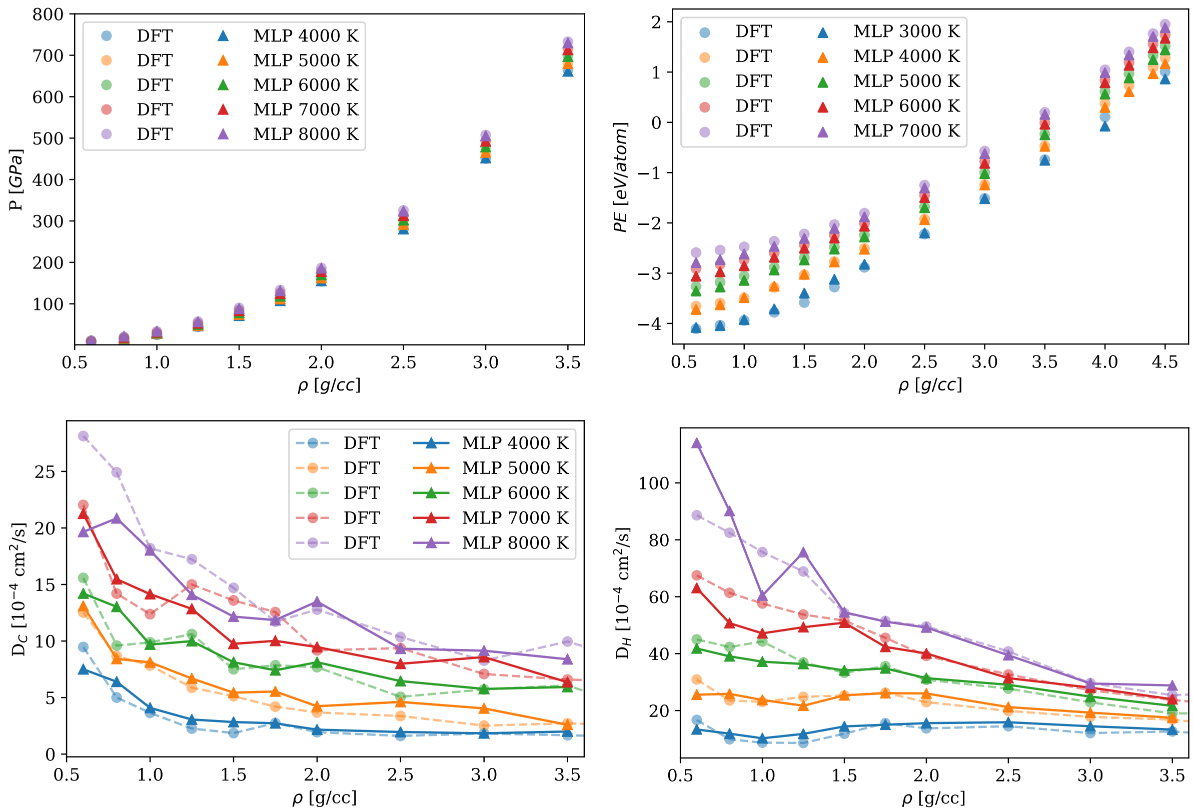

FIG. S9. Comparison between the potential energy (PE), pressure (P), the diffusion coefficient of hydrogen (D_H), and the diffusion coefficient of carbon (D_C), predicted by PBE DFT and the MLP. The results are from MD simulations of 54 CH_4 molecules in the NVT ensemble.

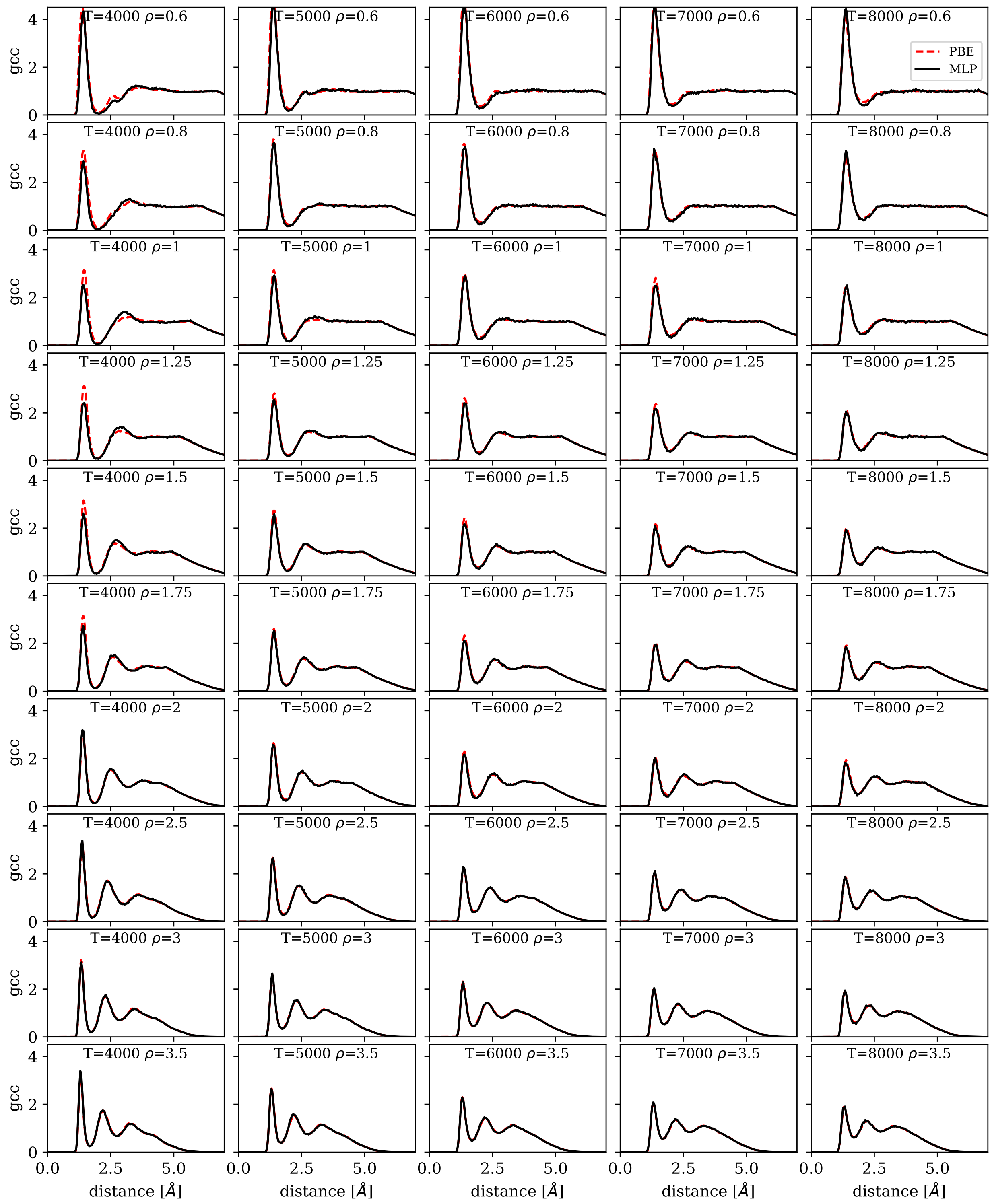

FIG. S10. Carbon-carbon radial distribution functions gcc of methane computed from equilibrium MD simulations in the NVT ensemble with a system size of 54 CH_4 formula units. The solid black lines are the results from the MLP, and the dashed red lines are from PBE DFT.

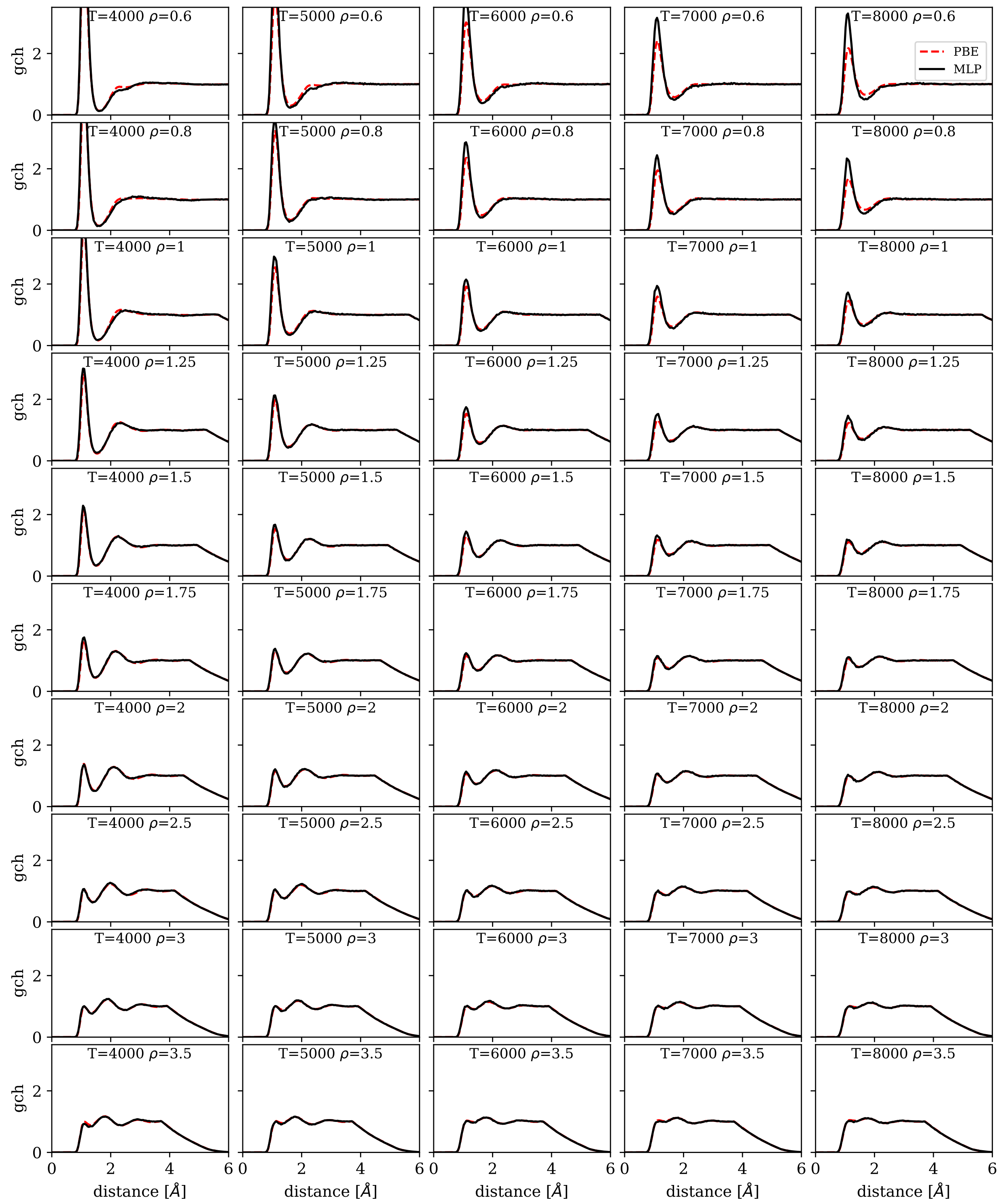

FIG. S11. Carbon-hydrogen radial distribution functions gch of methane computed from equilibrium MD simulations at the NVT ensemble with a system size of 54 CH_4 formula units. The solid black lines are the results from the MLP, and the dashed red lines are from PBE DFT.

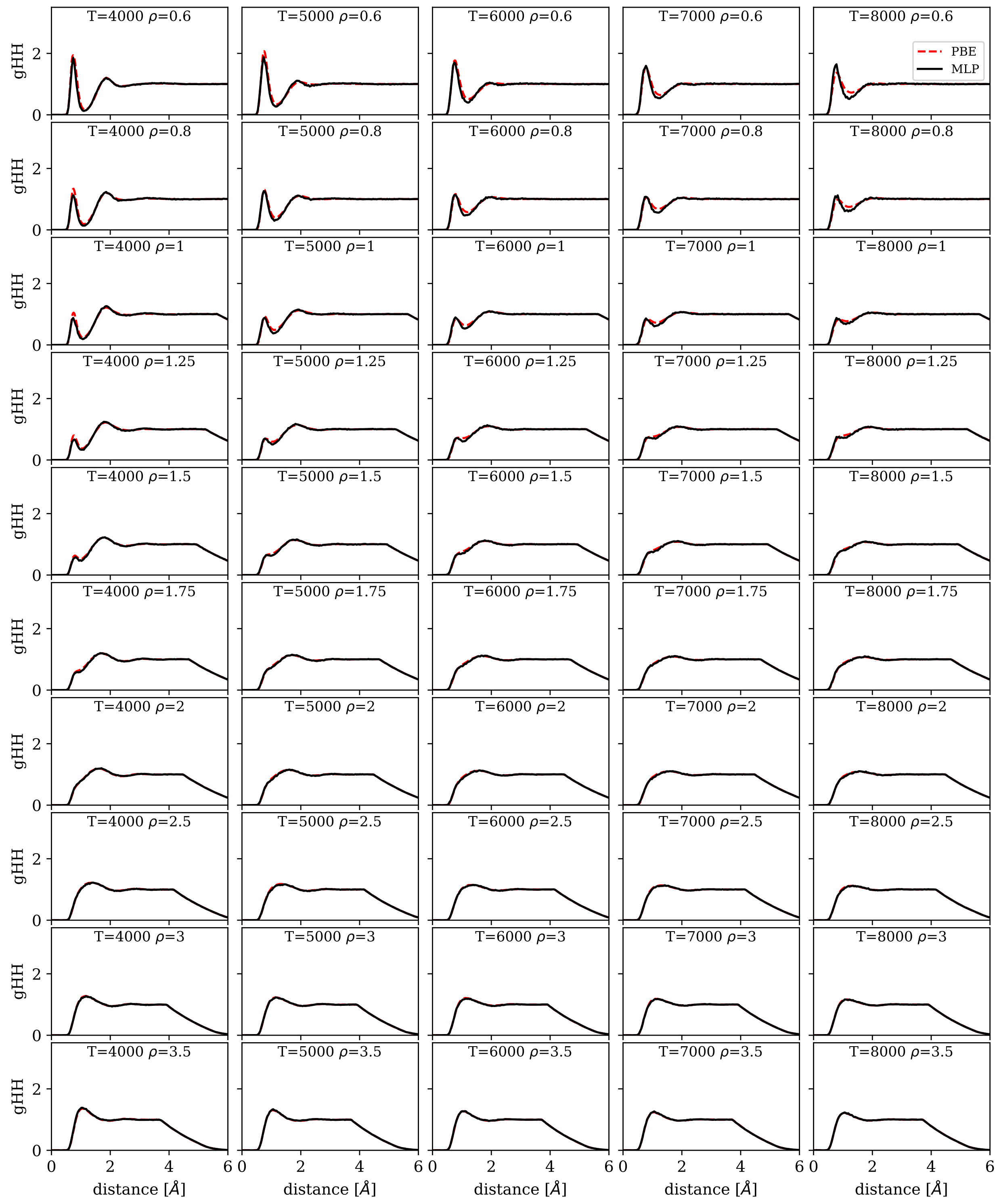

FIG. S12. Hydrogen-hydrogen radial distribution functions ghh of methane computed from equilibrium MD simulations at the NVT ensemble with a system size of 54 CH_4 formula units. The solid black lines are the results from the MLP, and the dashed red lines are from PBE DFT.

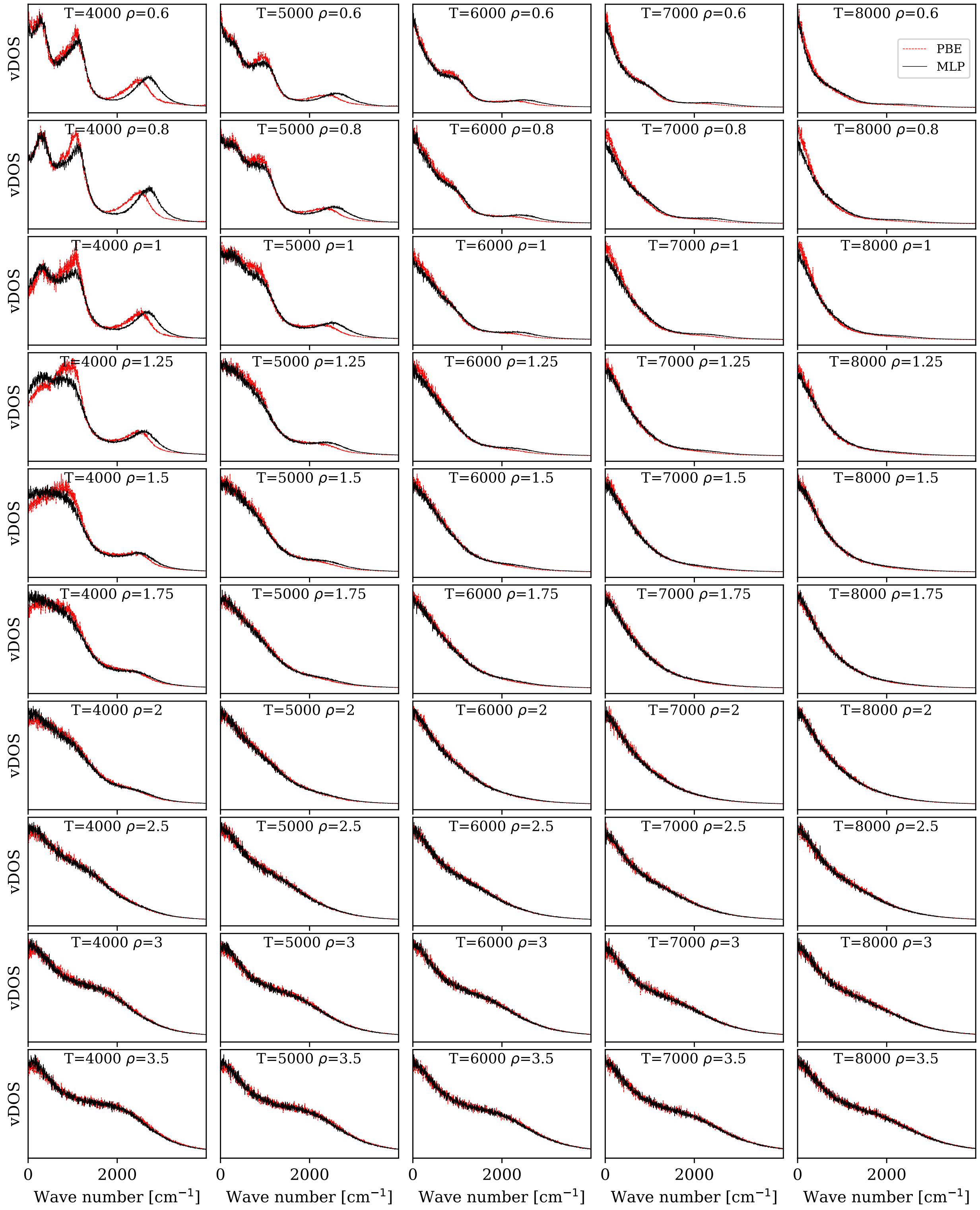

FIG. S13. Vibrational density of states (vDOS) of methane computed from equilibrium MD simulations in the NVT ensemble with a system size of 54 CH_4 formula units. The solid black lines are the results from the MLP, and the dashed red lines are from PBE DFT.

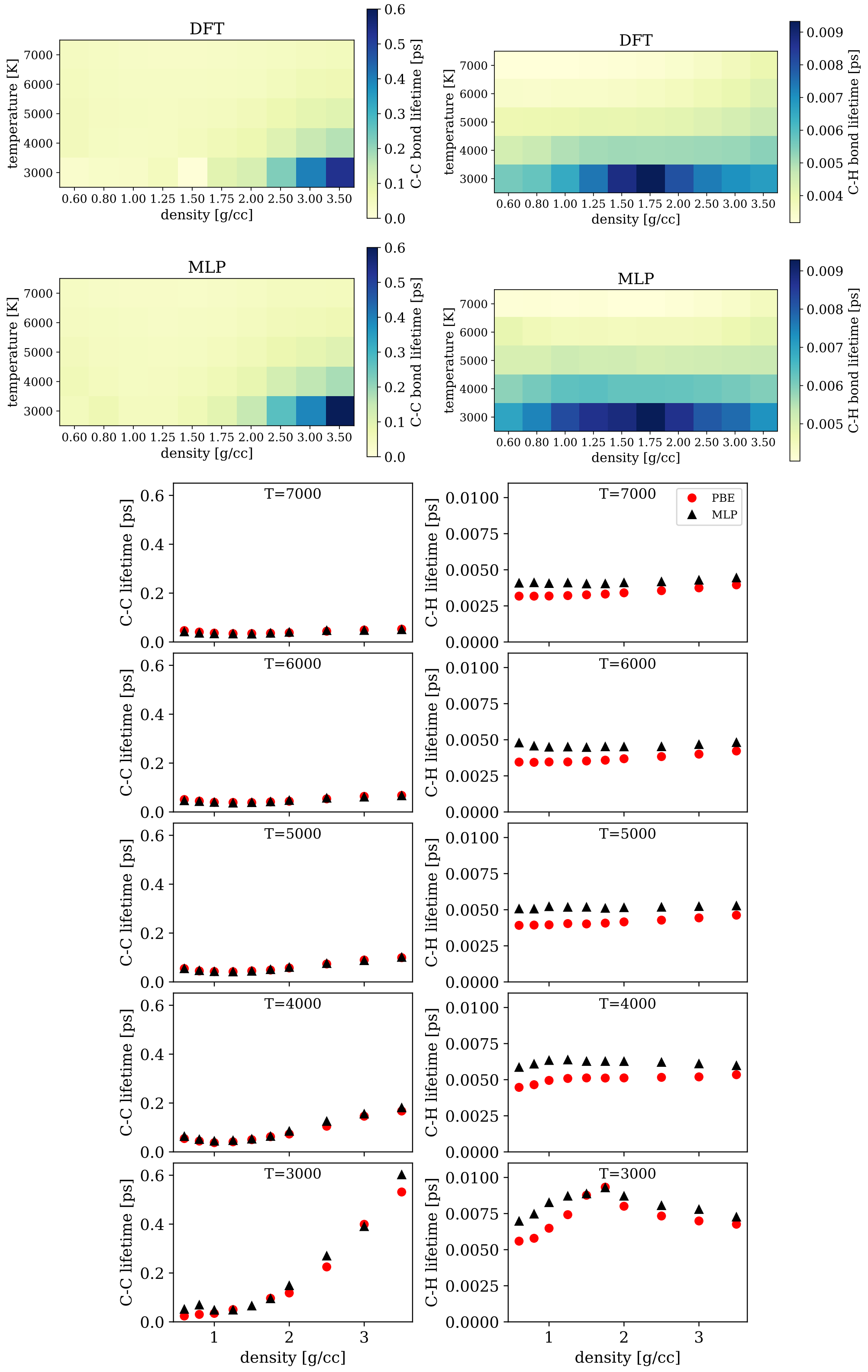

FIG. S14. Comparison between the C-C and C-H bond lifetimes predicted by PBE DFT and the MLP. The results are from MD simulations of 54 CH_4 molecules in the NVT ensemble.

E. NVT simulation of CH_2

We performed NVT simulations for systems of 48 CH_2 molecules using the MLP. The initial configuration resembles an amorphous structure. The equations of state are plotted in Fig. S15. The C-C, C-H and H-H radial distribution functions are plotted in Fig. S16, Fig. S17, and Fig. S18, respectively. The vibrational density of states are plotted in Fig. S19.

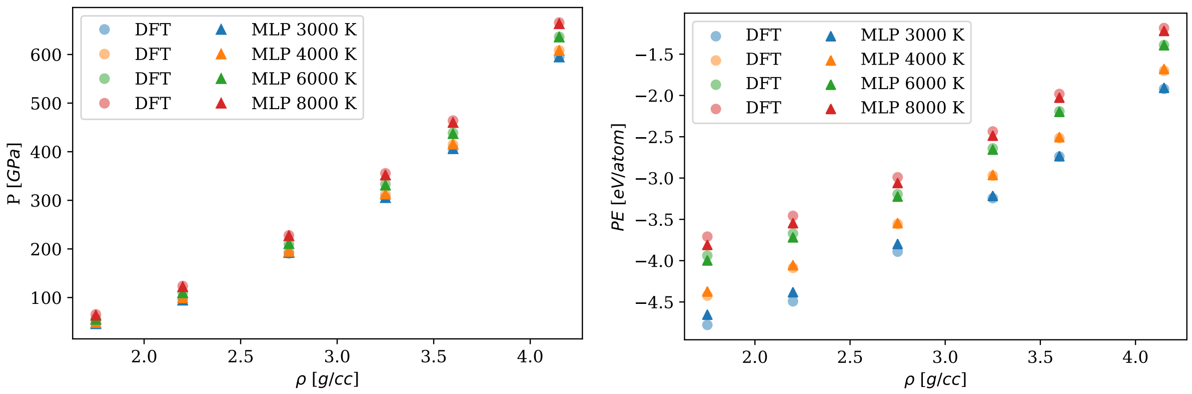

FIG. S15. Comparison between the potential energy (PE) and pressure (P) predicted by PBE DFT and the MLP. The results are from MD simulations of 48 CH_2 molecules in the NVT ensemble.

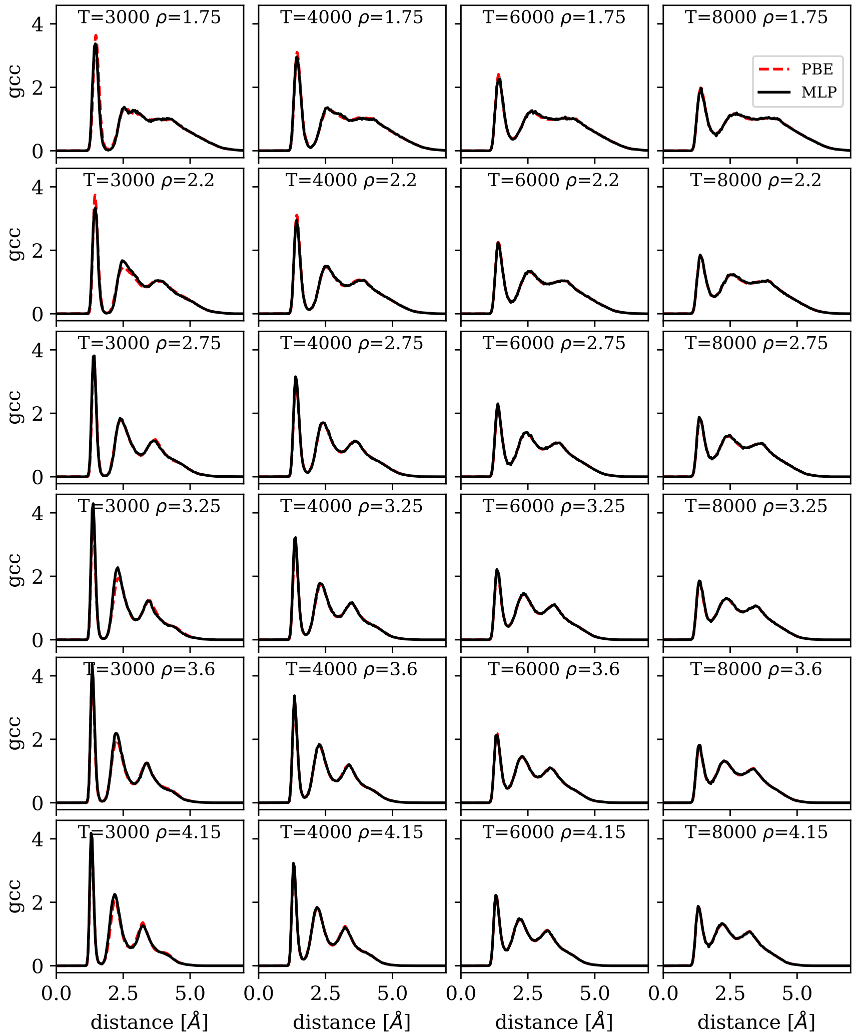

FIG. S16. Carbon-carbon radial distribution functions gcc of CH_2 computed from equilibrium MD simulations in the NVT ensemble with a system size of 48 CH_2 formula units. The solid black lines are the results from the MLP, and the dashed red lines are from PBE DFT.

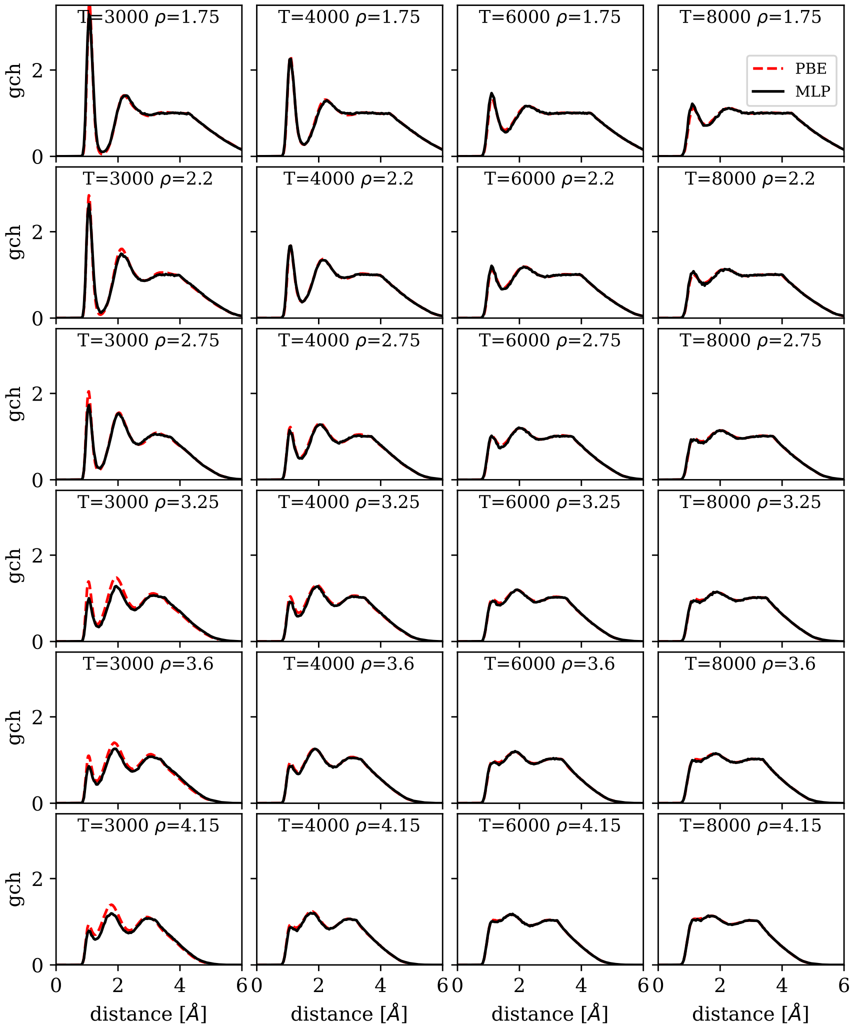

FIG. S17. Carbon-hydrogen radial distribution functions gch of CH_2 computed from equilibrium MD simulations at the NVT ensemble with a system size of 48 CH_2 formula units. The solid black lines are the results from the MLP, and the dashed red lines are from PBE DFT.

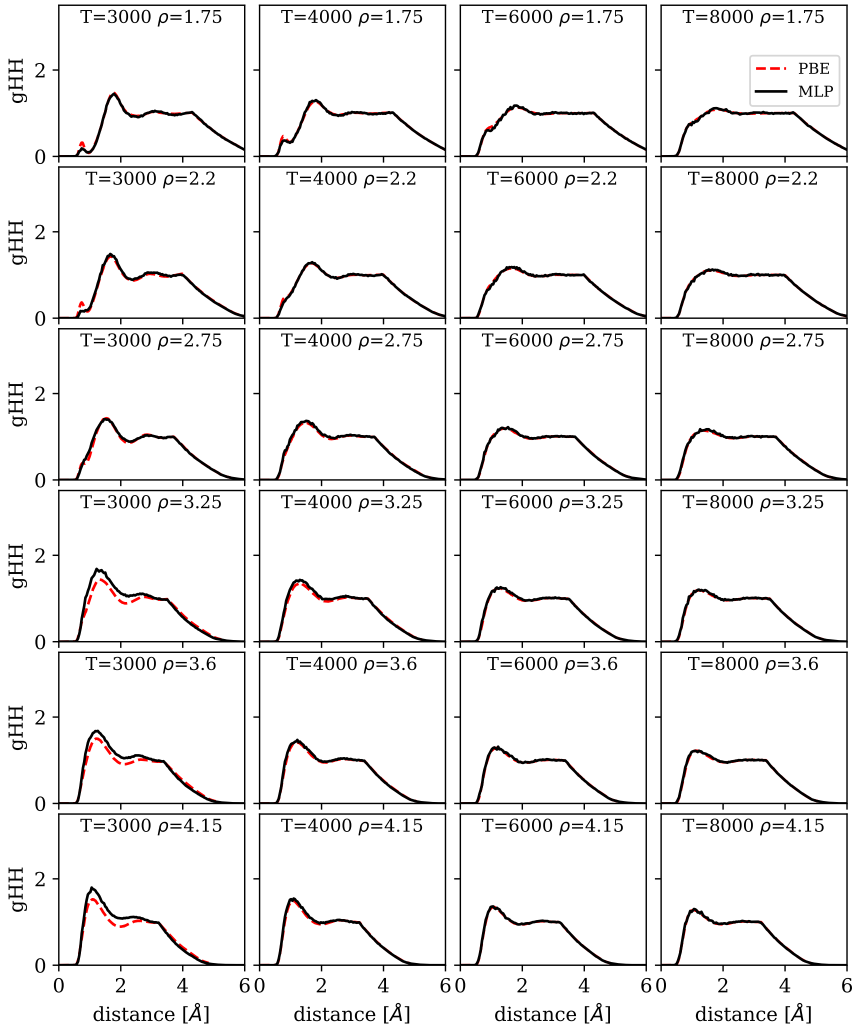

FIG. S18. Hydrogen-hydrogen radial distribution functions ghh of CH_2 computed from equilibrium MD simulations at the NVT ensemble with a system size of 48 CH_2 formula units. The solid black lines are the results from the MLP, and the dashed red lines are from PBE DFT.

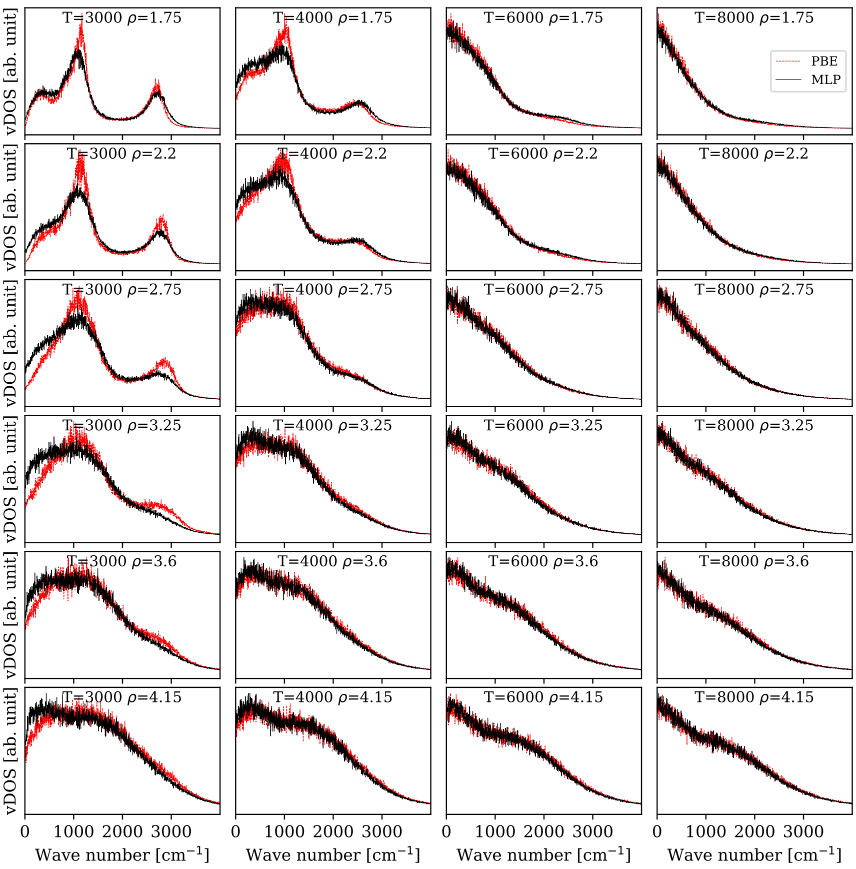

FIG. S19. Vibrational density of states (vDOS) of CH_2 computed from equilibrium MD simulations at the NVT ensemble with a system size of 48 CH_2 formula units. The solid black lines are the results from the MLP, and the dashed red lines are from PBE DFT.

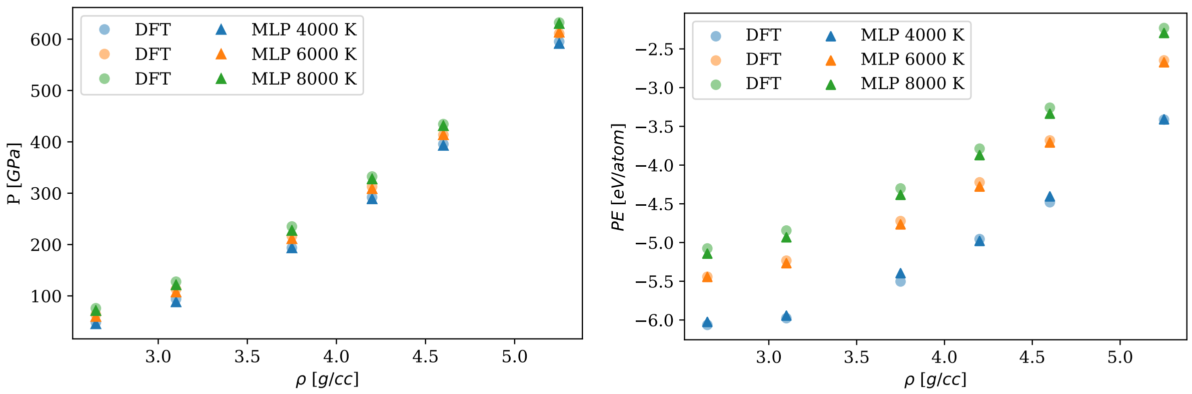

FIG. S20. Comparison between the potential energy (PE) and pressure (P) predicted by PBE DFT and the MLP. The results are from MD simulations of 24 C_2H molecules in the NVT ensemble.

F. NVT simulation of C_2H

We performed NVT simulations for systems of 24 C_2H molecules using the MLP. The initial configuration resembles an amorphous structure. The equations of state are plotted in Fig. S20. The C-C, C-H and H-H radial distribution functions are plotted in Fig. S21, Fig. S22, and Fig. S23, respectively. The vibrational density of states are plotted in Fig. S24.

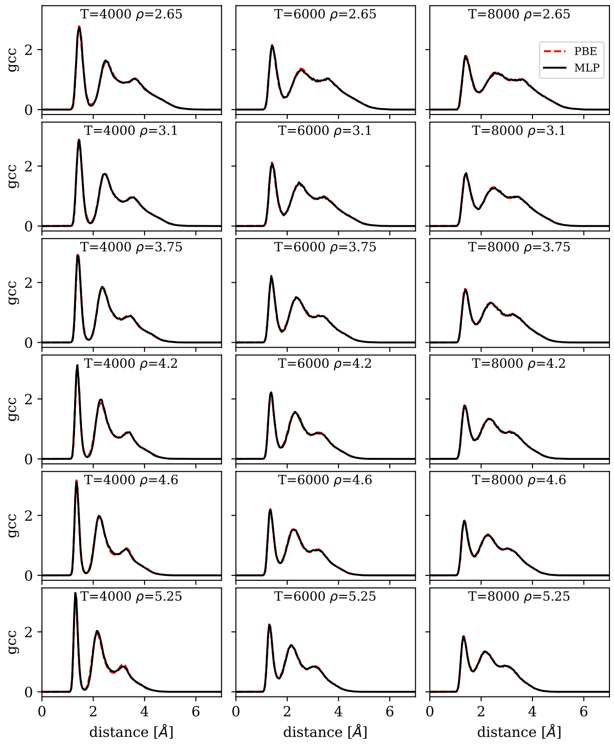

FIG. S21. Carbon-carbon radial distribution functions gcc of CH_2 computed from equilibrium MD simulations in the NVT ensemble with a system size of 24 C_2H formula units. The solid black lines are the results from the MLP, and the dashed red lines are from PBE DFT.

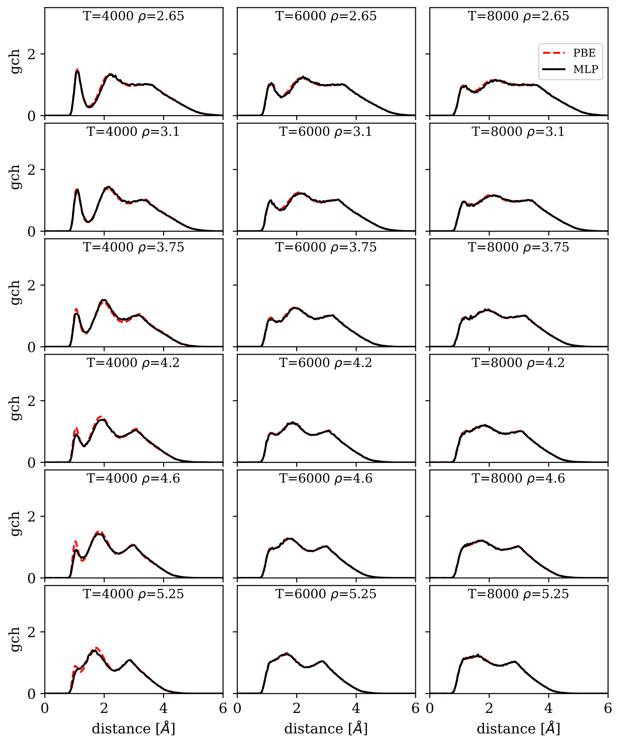

FIG. S22. Carbon-hydrogen radial distribution functions gch of C_2H computed from equilibrium MD simulations at the NVT ensemble with a system size of 24 C_2H formula units. The solid black lines are the results from the MLP, and the dashed red lines are from PBE DFT.

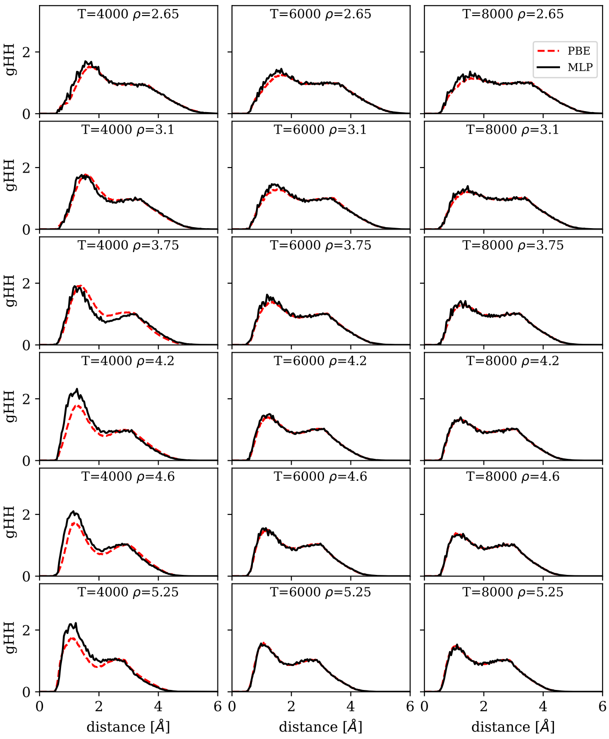

FIG. S23. Hydrogen-hydrogen radial distribution functions ghh of C_2H computed from equilibrium MD simulations at the NVT ensemble with a system size of 24 C_2H formula units. The solid black lines are the results from the MLP, and the dashed red lines are from PBE DFT.

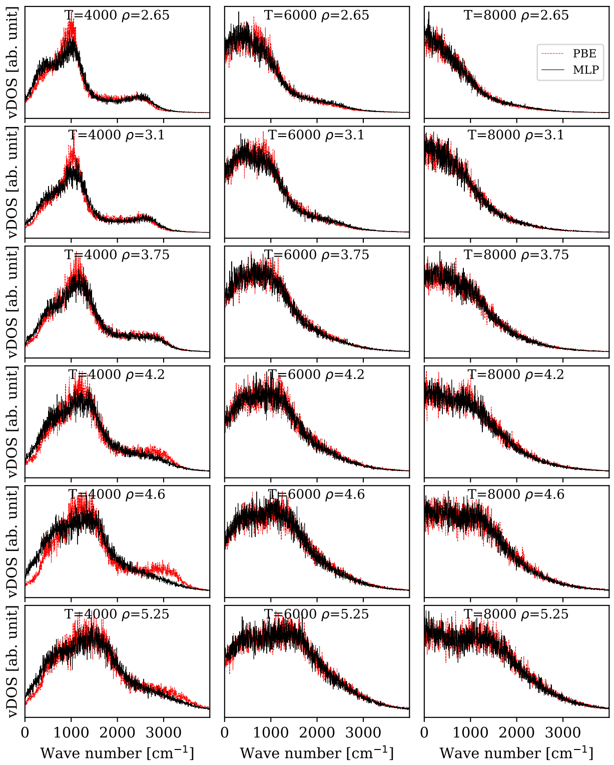

FIG. S24. Vibrational density of states (vDOS) of C_2H computed from equilibrium MD simulations at the NVT ensemble with a system size of 24 C_2H formula units. The solid black lines are the results from the MLP, and the dashed red lines are from PBE DFT.

G. Enthalpy of different solid carbon polymorphs

We first performed random structure searches [20] using the MLP to find low-energy solid carbon polymorphs. Using these polymorphs, we computed the 0 K enthalpy at pressures from 0 to 900 GPa, employing both the MLP and PBE DFT. The results are shown in Fig. S25. The Fddd phase is graphite, while the Fd3m, R3m, and P63mmc phases are diamond structures with different stacking sequences. Ia3 structure is also often referred to as BC8 carbon.

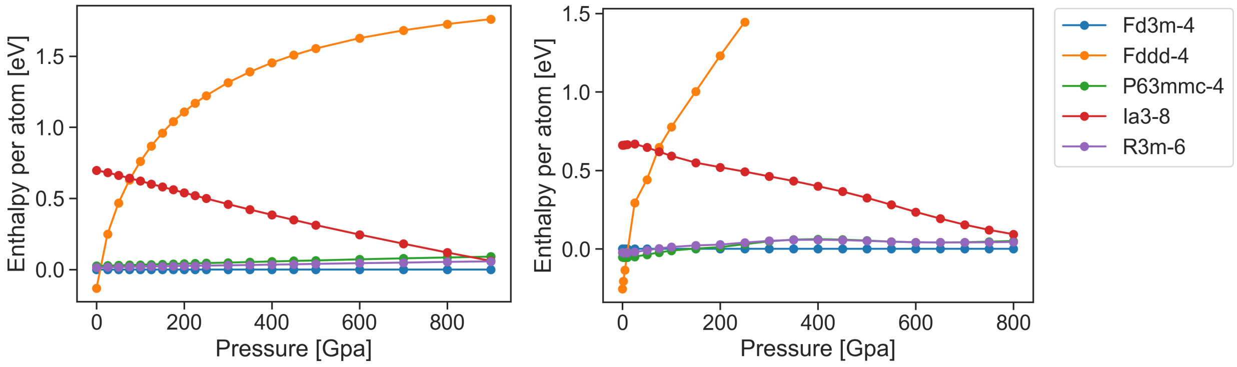

FIG. S25. 0 K enthalpy curves for different solid carbon phases. The left is from PBE DFT, and the right is from MLP.

V. SIMULATIONS USING THE MLP

A. MLP MD simulations

All MLP MD simulations were performed in LAMMPS [21] with a neural network potential implementation [22]. The simulations of the bulk phases were performed in the NPT ensemble using the Nosé-Hoover isotropic barostat. The time step size was chosen to 0.25 fs for C/H mixtures, 0.4 fs for pure carbon, and 0.2 fs for pure hydrogen.

B. NPT simulations of diamond and pure liquid carbon

A series of NPT simulations was performed at 2500 K to 10000 K and 10 GPa to 600 GPa for diamond and pure liquid carbon phases. The system size was 512 carbon atoms. The simulation time was 120 ps. Besides computing the EOS, we collected the diffusion coefficient of carbon in the bulk liquid (Fig. S26).

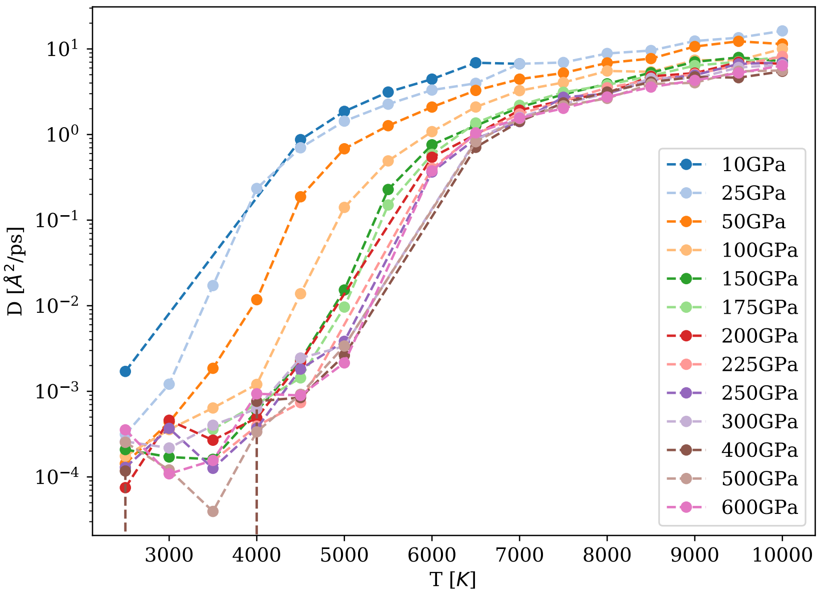

FIG. S26. Diffusion coefficient of carbon in the bulk liquid computed using the MLP in the NPT ensemble.

C. Chemical potentials of pure carbon systems

To compute $\Delta\mu_D$, interface pinning simulations [23] were performed using the PLUMED code [24] on a diamond-liquid carbon system containing 1,024 carbon atoms at pressures between 0 GPa and 800 GPa employing the MLP. The simulation length was 80 ps after equilibration. A Nosé-Hoover barostat was only applied along the z direction, which is perpendicular to the interface in the coexistence simulations. The dimensions of the supercell along x and y directions were commensurate with the equilibrium lattice parameters of the diamond phase at the given conditions. An umbrella potential with stiffness α was added to the Hamiltonian of the system:

$$\mathcal{H}_{biased}(\mathbf{q}) = \mathcal{H}(\mathbf{q}) + \frac{\alpha}{2}\left(\Phi - \bar{\Phi}\right)^2, \quad (4)$$

where $\Phi = \sum_{i=1}^{N} \phi_i$ is an extensive collective variable (CV) of the whole system that is constructed by summing up the order parameters ϕ_i of each carbon atom. More specifically, we used a locally-averaged [25] Q_3 order parameter [26] as atom-centered order parameter for detecting diamond structures, which we transformed with a hyperbolic switching function to enhance its resolution capability between solid and liquid-like atomic environments [27]. The relevant part of the PLUMED input files that contain the specification of the order parameters are:

```
Q3 ...
LABEL=q3
SPECIES=1-1024 SWITCH={CUBIC D_0=1.6 D_MAX=1.8}
MEAN
LOWMEM
... Q3

LOCAL_AVERAGE ...
LABEL=m3
SPECIES=q3
SWITCH={CUBIC D_0=1.6 D_MAX=1.8}
MEAN
MORE_THAN1={SMAP R_0=0.05 D_0=0.2 A=8 B=8}
LOWMEM
... LOCAL_AVERAGE
```

```
# apply the umbrella potential
RESTRAINT ARG=m3.morethan-1 AT=250 KAPPA=1.0 LABEL=res
```

Finally, the difference in chemical potential per atom between the diamond and the liquid carbon phases in the simulations can be computed using

$$\Delta\mu_D = \mu_{diamond} - \mu_{liquidC} = \alpha\frac{\bar{\Phi} - \langle\Phi\rangle}{\langle\phi\rangle_{diamond} - \langle\phi\rangle_{liquidC}}, \tag{5}$$

where $\langle\Phi\rangle$ is the average order parameter from the interface pinning simulations, and $\langle\phi\rangle_{diamond}$ and $\langle\phi\rangle_{liquidC}$ are the average values of the atomic order parameter of carbon in the pure phases at the same conditions. The computed relative chemical potentials from the interface pinning calculations are shown in Fig. S27. To extend the chemical potentials to a wide range of pressures and temperatures, we used the thermodynamic integration method [28, 29], by numerically integrating the Gibbs–Duhem relation along isotherms and the Gibbs–Helmholtz relations along isobars, respectively.

D. Distribution of C-C bonds in CH_4

As discussed in the main text, we analyzed the average numbers and the lifetimes of the C-C and the C-H bonds in the CH_4 composition over a wide range of thermodynamic conditions. In Fig. S28, we show the distribution for the number of C-C bonds that each carbon atom has. It can be seen that, at all conditions considered, the distribution is rather broad than sharply peaked at a specific bond number. In other words, if on average each carbon atom has 1 bond, this means some C atoms have one bond, some have two bonds, some have zero bond, and so on.

E. Chemical potential of C in C/H mixtures

The S0 method for computing the chemical potentials in a mixture only uses equilibrium MD NPT simulations of C/H mixtures with carbon fraction χ_C. In practice, we started with a pure carbon system of 64 C atoms, adding different amounts of hydrogen atoms (1, 2, 4, 6, 8, 12, 16, 24, 32, 40, 48, 56, 64, 72, 80, 88, 96, 104, 112, 128, 144, 160, 192, 224, 256, 288, 352, 384, 448, 512, 672, 768, 896, 1024, 1280, 1536, 2048, 2560, 3072), and then replicated the simulation box

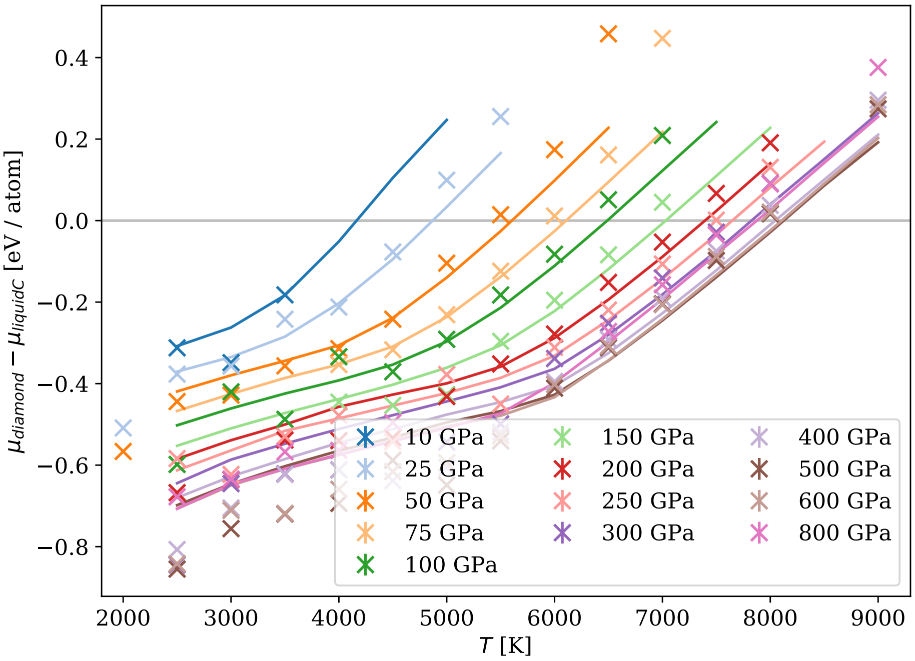

FIG. S27. The relative chemical potentials of diamond shown relative to the chemical potential of liquid carbon at each indicated pressure and temperature, computed at the MLP level in the interface pinning simulations. The standard mean errors are indicated using the error bars in **a**-**f**. The error bars are smaller than the symbols. The solid lines in are not fits, but independently calculated from thermodynamic integration calculations using the MLP.

a few times in all dimensions such that the total number of atoms are roughly between 10,000 and 100,000. For each run, the simulation length was about 10 ps.

In Fig. S29 we show a snapshot from a MD run, where the liquid-liquid phase separation (PT2) can be observed. A movie of the PT2 is also provided in the Supplementary Information.

Interface pinning simulations [30, 31] were performed on a diamond–C/H liquid coexistence system containing 1,024 C atoms and varying number of H atoms (ranging from 128 to 2,560) at pressures between 0 GPa and 600 GPa. The simulation time was about 50 ps. A snapshot of the coexistence system is in the Fig. S30. The simulations are performed using umbrella sampling based on the locally averaged Q3 order parameter as described before.

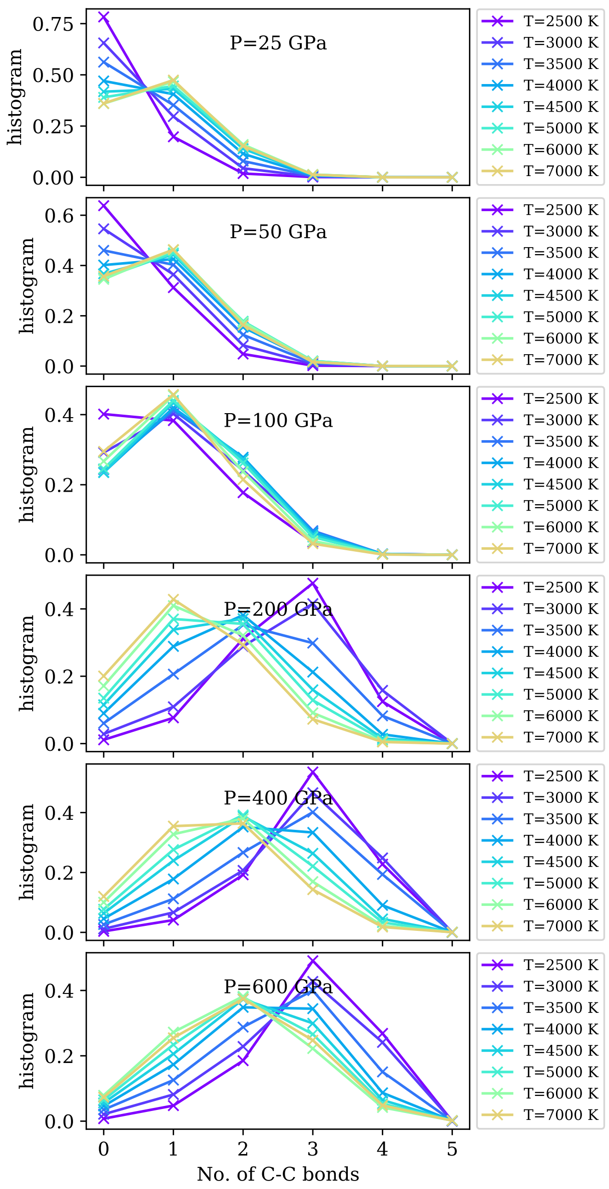

FIG. S28. The distribution of the C-C bonds that each carbon atom has in the CH4 system at different conditions.

F. Hydrocarbon crystals

The C/H system can form a large variety of crystal structures, which have been probed from DFT crystal structure searches [32–36]. The current understanding is that CH_4 will decompose into diamond and hydrogen at 0 K and pressures above 300 GPa (considering zero-point energies) [34, 35], CH_2 and CH can form diamond and lighter C/H crystals at 0 K and pressures above about 20 GPa (considering zero-point energies) [35]. For finite-temperature free energies, harmonic

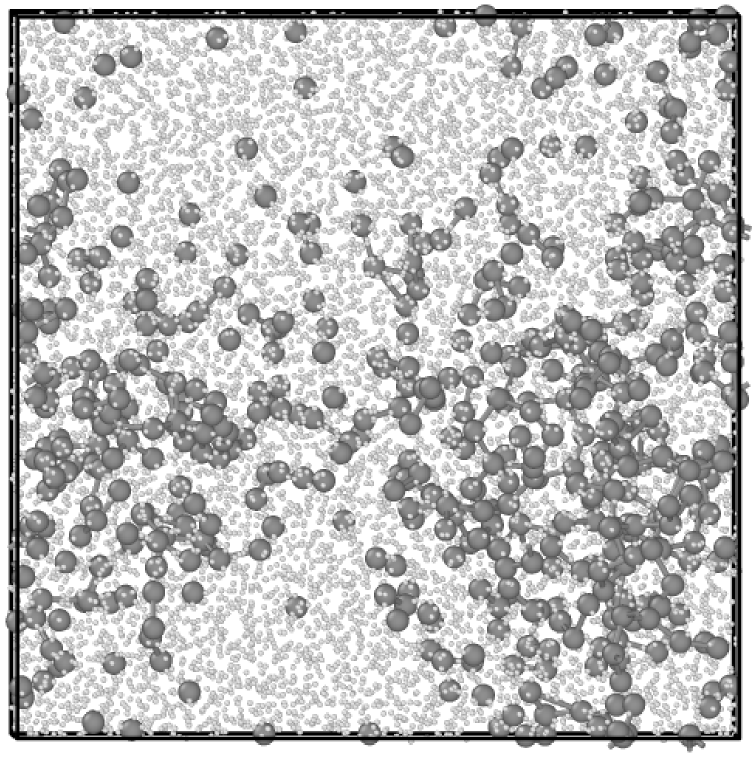

FIG. S29. A snapshot of the atomic coordinates from MD simulations at P=400 GPa, T=3000 K. The system contains 512 carbon atoms and 10,240 hydrogen atoms. Carbon atoms are shown as gray spheres, and hydrogen atoms are shown as white spheres.

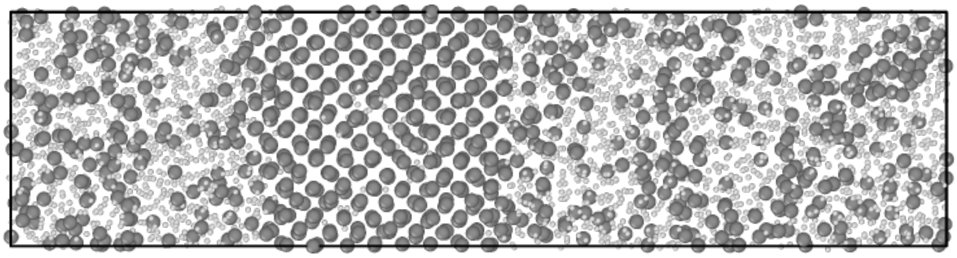

FIG. S30. An illustration of the simulation setup used in the coexistence simulations for computing the chemical potential difference between diamond and the carbon atoms dissolved in the C/H mixture. Carbon atoms are shown as gray spheres, and hydrogen atoms are shown as white spheres.

approximation suggests that lattice vibrations massively stabilize the diamond phase, making it the most stable composition at temperatures high than about 1000 K-3000 K [34]. Furthermore, the melting point of CH_4 crystals is between 1000 K and 1500 K. Indeed, in our DFT MD and MLP MD simulations, CH_4 crystals melt quickly at $T \geq 2000$ K. This means that the C/H crystals are less stable than either liquid or diamond at the temperature range considered for diamond formation in this work ($T \geq 3000$ K). As such, the possible formation of C/H crystals are not relevant in the current study.

VI. CARBON CONTENTS OF PLANETS AND STARS

We highlight here a few examples of carbon and hydrogen mixtures found in giant planets and carbon-rich stars.

A. Atmosphere

The measured elemental abundance in the atmosphere of Neptune and Uranus is taken from Table 3 in Ref. [37]. To go from the ratios to concentrations we assume that these elements are the totality of elements present, i.e. $[C] + [He] + [S] + [H] = 1$, divide each side by the concentration of hydrogen to get $[C]/[H] + [He]/[H] + [S]/[H] + 1 = 1/[H]$ which, given the measured abundance ratios, can be solved to get the concentration of hydrogen and other elements. For Uranus the elemental ratios are: $He/H = 0.09 \pm 0.02$, $C/H = 0.0236 \pm 0.003$ and $S/H = 0.00032 \pm 0.00016$. Hence,

$[C] + [He] + [S] + [H] = 1$

$0.0236 + 0.09 + 0.00032 + 1 = 1/[H]$

$[H] = 0.8977$

$[C]/[H] = 0.0236; [C] = 0.0212$

$[He]/[H] = 0.09; [He] = 0.0808$

$[S]/[H] = 0.00032; [S] = 0.0003$

Measured elemental abundance ratios in Neptune are similar to Uranus: $He/H = 0.117 \pm 0.02$, $C/H = 0.0247 \pm 0.0062$ and $S/H = 0.00032 \pm 0.00016$. Concentrations are:

$[C] + [He] + [S] + [H] = 1$

$0.0247 + 0.117 + 0.00032 + 1 = 1/[H]$

$[H] = 0.8756$

$[C]/[H] = 0.0247; [C] = 0.0216$

$[He]/[H] = 0.117; [He] = 0.1025$

$[S]/[H] = 0.00032; [S] = 0.0003$

The concentration of carbon in both planets is about 2%.

B. Neptune and Uranus interior models

Elemental abundances in the interior of the ice giant planets are model dependent, since they are generally not accessible to direct measurements. Recent Uranus models by Nettelmann et al. [38] use equations of state for CH_4, NH_3 and H_2O that are linearly mixed in the inner envelope with (CH_4:NH_3:H_2O) proportions of (4:1:7.7). In terms of elemental ratios this is equivalent to $C/H = 0.1162$, $N/H = 0.0291$ and $O/H = 0.2238$. Concentration are:

$[C] + [N] + [O] + [H] = 1$

$0.1162 + 0.0291 + 0.2238 + 1 = 1/[H]$

$[H] = 0.7304$

$[C]/[H] = 0.1162; [C] = 0.0849$

$[N]/[H] = 0.0291; [N] = 0.0213$

$[O]/[H] = 0.2238; [O] = 0.1635$

The concentration of carbon is about 8% in these interior models with a pressure range between 10 GPa and 550 GPa for the inner envelope.

C. White Dwarfs

Recently, Hollands *et al.* reported an example of a White Dwarf star with a mixed carbon-hydrogen atmosphere with a C/H ratio of 0.15 [39]. Helium was not detected in the spectra hence the carbon and hydrogen concentrations are 13% and 87% respectively. Deeper in the star the composition is thought to be a mixture of carbon, oxygen and neon.

[1] G. Kresse and J. Hafner, "Ab initio molecular dynamics for liquid metals," Phys. Rev. B **47**, 558 (1993).

[2] G. Kresse and J. Hafner, "Ab initio molecular-dynamics simulation of the liquid-metal-amorphous-semiconductor transition in germanium," Phys. Rev. B **49**, 14251 (1994).

[3] G. Kresse and J. Furthmüller, "Efficient iterative schemes for ab initio total-energy calculations using a plane-wave basis set," Phys. Rev. B **54**, 11169 (1996).

[4] J. P. Perdew, K. Burke, and M. Ernzerhof, "Generalized gradient approximation made simple," Phys. Rev. Lett. **77**, 3865 (1996).

[5] L. X. Benedict, K. P. Driver, S. Hamel, B. Militzer, T. Qi, A. A. Correa, A. Saul, and E. Schwegler, "Multiphase equation of state for carbon addressing high pressures and temperatures," Phys. Rev. B **89**, 224109 (2014).

[6] K. Ramakrishna, T. Dornheim, and J. Vorberger, "Influence of finite temperature exchange-correlation effects in hydrogen," Phys. Rev. B **101**, 195129 (2020).

[7] M. Bonitz, T. Dornheim, Z. A. Moldabekov, S. Zhang, P. Hamann, H. Kählert, A. Filinov, K. Ramakrishna, and J. Vorberger, "Ab initio simulation of warm dense matter," Physics of Plasmas **27**, 042710 (2020), https://doi.org/10.1063/1.5143225.

[8] M. D. Knudson and M. P. Desjarlais, "Interplay of high-precision shock wave experiments with first-principles theory to explore molecular systems at extreme conditions: A perspective," Journal of Applied Physics **129**, 210904 (2021), https://doi.org/10.1063/5.0050878.

[9] M. Millot, S. Hamel, J. R. Rygg, P. M. Celliers, G. W. Collins, F. Coppari, D. E. Fratanduono, R. Jeanloz, D. C. Swift, and J. H. Eggert, "Experimental evidence for superionic water ice using shock compression," Nature Physics **14**, 297 (2018).

[10] Y.-J. Kim, B. Militzer, B. Boates, S. Bonev, P. M. Celliers, G. W. Collins, K. P. Driver, D. E. Fratanduono, S. Hamel, R. Jeanloz, J. R. Rygg, D. C. Swift, J. H. Eggert, and M. Millot, "Evidence for dissociation and ionization in shock compressed nitrogen to 800 gpa," Phys. Rev. Lett. **129**, 015701 (2022).

[11] M. French, T. R. Mattsson, N. Nettelmann, and R. Redmer, "Equation of state and phase diagram of water at ultrahigh pressures as in planetary interiors," Phys. Rev. B **79**, 054107 (2009).

[12] S. Nosé, "A unified formulation of the constant temperature molecular-dynamics methods," J. Chem. Phys. **81**, 511 (1984).

[13] M. Bethkenhagen, E. R. Meyer, S. Hamel, N. Nettelmann, M. French, L. Scheibe, C. Ticknor, L. A. Collins, J. D. Kress, J. J. Fortney, and R. Redmer, "Planetary ices and the linear mixing approximation," Astrophys. J. **848**, 67 (2017).

[14] J. Behler and M. Parrinello, "Generalized neural-network representation of high-dimensional potential-energy surfaces," Phys. Rev. Lett. **98**, 146401 (2007).

[15] A. Singraber, T. Morawietz, J. Behler, and C. Dellago, "Parallel multistream training of high-dimensional neural network potentials," Journal of Chemical Theory and Computation **15**, 3075 (2019).

[16] P. Rowe, V. L. Deringer, P. Gasparotto, G. Csányi, and A. Michaelides, "An accurate and transferable machine learning potential for carbon," J. Chem. Phys. **153**, 034702 (2020).

[17] V. L. Deringer and G. Csányi, "Machine learning based interatomic potential for amorphous carbon," Physical Review B **95** (2017), 10.1103/physrevb.95.094203.

[18] B. Cheng, R.-R. Griffiths, S. Wengert, C. Kunkel, T. Stenczel, B. Zhu, V. L. Deringer, N. Bernstein, J. T. Margraf, K. Reuter, *et al.*, "Mapping materials and molecules," Accounts of Chemical Research **53**, 1981 (2020).

[19] B. Cheng, G. Mazzola, C. J. Pickard, and M. Ceriotti, "Evidence for supercritical behaviour of high-pressure liquid hydrogen," Nature **585**, 217 (2020).

[20] C. J. Pickard and R. J. Needs, "Ab initio random structure searching," Journal of Physics: Condensed Matter **23**, 053201 (2011).

[21] S. Plimpton, "Fast Parallel Algorithms for Short-Range Molecular Dynamics," J. Comput. Phys. **117**, 1 (1995).

[22] A. Singraber, J. Behler, and C. Dellago, "Library-based LAMMPS implementation of high-dimensional neural network potentials," J. Chem. Theory Comput. **15**, 1827 (2019).

[23] U. R. Pedersen, F. Hummel, G. Kresse, G. Kahl, and C. Dellago, "Computing gibbs free energy differences by interface pinning," Phys. Rev. B **88**, 94101 (2013).

[24] G. A. Tribello, M. Bonomi, D. Branduardi, C. Camilloni, and G. Bussi, "Plumed 2: New feathers for an old bird," Comput. Phys. Commun **185**, 604 (2014).

[25] W. Lechner and C. Dellago, "Accurate determination of crystal structures based on averaged local bond order parameters," J. Chem. Phys. **129**, 114707 (2008).

[26] P. J. Steinhardt, D. R. Nelson, and M. Ronchetti, "Bond-orientational order in liquids and glasses," Physical Review B **28**, 784 (1983).

[27] B. Cheng, G. A. Tribello, and M. Ceriotti, "Solid-liquid interfacial free energy out of equilibrium," Phys. Rev. B **92**, 180102 (2015).

[28] B. Cheng and M. Ceriotti, "Computing the absolute Gibbs free energy in atomistic simulations: Applications to defects in solids," Phys. Rev. B **97**, 054102 (2018).

[29] A. Reinhardt and B. Cheng, "Quantum-mechanical exploration of the phase diagram of water," Nat. Commun. **12**, 1 (2021).

[30] U. R. Pedersen, F. Hummel, and C. Dellago, "Computing the crystal growth rate by the interface pinning method," J. Chem. Phys. **142**, 44104 (2015).

[31] B. Cheng, C. Dellago, and M. Ceriotti, "Theoretical prediction of the homogeneous ice nucleation rate: Disentangling thermodynamics and kinetics," Phys. Chem. Chem. Phys. **20**, 28732 (2018).

[32] G. Gao, A. R. Oganov, Y. Ma, H. Wang, P. Li, Y. Li, T. Iitaka, and G. Zou, "Dissociation of methane under high pressure," J. Chem. Phys. **133**, 144508 (2010).

[33] H. Liu, I. I. Naumov, and R. J. Hemley, "Dense hydrocarbon structures at megabar pressures," J. Phys. Chem. Lett. **7**, 4218 (2016).

[34] A. S. Naumova, S. V. Lepeshkin, and A. R. Oganov, "Hydrocarbons under pressure: Phase diagrams and surprising new compounds in the c-h system," J. Phys. Chem. C **123**, 20497 (2019).

[35] L. J. Conway and A. Hermann, "High pressure hydrocarbons revisited: from van der Waals compounds to diamond," Geosciences **9**, 227 (2019).

[36] T. Ishikawa and T. Miyake, "Evolutionary construction of a formation-energy convex hull: Practical scheme and application to a carbon-hydrogen binary system," Phys. Rev. B **101**, 214106 (2020).

[37] T. Guillot and D. Gautier, "10.16 - giant planets," in *Treatise on Geophysics (Second Edition)*, edited by G. Schubert (2015) second edition ed., pp. 529–557.

[38] N. Nettelmann, K. Wang, J. J. Fortney, S. Hamel, S. Yellamilli, M. Bethkenhagen, and R. Redmer, "Uranus evolution models with simple thermal boundary layers," Icarus **275**, 107 (2016).

[39] M. A. Hollands, P. E. Tremblay, B. T. Gänsicke, M. E. Camisassa, D. Koester, A. Aungwerojwit, P. Chote, A. H. Córsico, V. S. Dhillon, N. P. Gentile-Fusillo, M. J. Hoskin, P. Izquierdo, T. R. Marsh, and D. Steeghs, "An ultra-massive white dwarf with a mixed hydrogen–carbon atmosphere as a likely merger remnant," Nature Astronomy **4**, 663 (2020).
