# Supplementary material for: Thermodynamics of diamond formation from hydrocarbon mixtures in planets
Source: Nat Commun. 2023 Feb 27;14:1104. doi: 10.1038/s41467-023-36841-1 (PMC9968715; doi:10.1038/s41467-023-36841-1)
Supplement: Supplementary file 2 — Description of Additional Supplementary Files [file 41467_2023_36841_MOESM2_ESM.pdf]

Description of Additional Supplementary Files

Supplementary Movie 1: Liquid-liquid phase separation (PS2) of high-pressure C/H mixture
